# Simple transformations capture auditory input to cortex

**DOI:** 10.1073/pnas.1922033117

**Published:** 2020-10-23

**Authors:** Monzilur Rahman, Ben D. B. Willmore, Andrew J. King, Nicol S. Harper

**Affiliations:** ^a^Department of Physiology, Anatomy and Genetics, University of Oxford, OX1 3PT Oxford, United Kingdom

**Keywords:** encoding models of neural responses, models of the auditory periphery, auditory cortex, predicting responses to natural sounds, Marr’s levels of analysis

## Abstract

Sounds are processed by the ear and central auditory pathway. These processing steps are biologically complex, and many aspects of the transformation from sound waveforms to cortical response remain unclear. To understand this transformation, we combined models of the auditory periphery with various encoding models to predict auditory cortical responses to natural sounds. The cochlear models ranged from detailed biophysical simulations of the cochlea and auditory nerve to simple spectrogram-like approximations of the information processing in these structures. For three different stimulus sets, we tested the capacity of these models to predict the time course of single-unit neural responses recorded in ferret primary auditory cortex. We found that simple models based on a log-spaced spectrogram with approximately logarithmic compression perform similarly to the best-performing biophysically detailed models of the auditory periphery, and more consistently well over diverse natural and synthetic sounds. Furthermore, we demonstrated that including approximations of the three categories of auditory nerve fiber in these simple models can substantially improve prediction, particularly when combined with a network encoding model. Our findings imply that the properties of the auditory periphery and central pathway may together result in a simpler than expected functional transformation from ear to cortex. Thus, much of the detailed biological complexity seen in the auditory periphery does not appear to be important for understanding the cortical representation of sound.

## Significance

Sensory systems are extremely complex, with diverse neurons and connections. However, this does not necessarily imply that the computations performed by these systems are also as complex. Here we examine the impact of processing in the ear and subcortical pathway on neural responses to natural sounds in the auditory cortex. We find that this can be described more consistently using simple spectral models. This suggests that there may be an underlying simplicity to the signal transformation from ear to cortex that is hidden among the detail. This hidden simplicity may be a feature of other sensory systems too.

Sensory systems, from the sense organs up through the neural pathway, are typically very complex, comprising many different structures and cell types that often interact in a nonlinear fashion. The complexity of these dynamic systems can make understanding their computations challenging. However, much of this physiological complexity may reflect biological constraints or come into play only under unusual conditions. Consequently, it could be that the signal transformations that they commonly compute are substantially simpler than their physical implementations (1). Taking the auditory system as an example, we aimed to empirically determine the computational transformation of auditory signals through the ear to the cortex. To understand this transformation, we appended various models of the auditory periphery to neural encoding models to predict auditory cortical responses to diverse sounds. We used both synthetic and natural sounds, as the latter are central to the normal function of the auditory pathway.

Various models of the auditory periphery have been developed and refined (2–11) to account for experimental observations of cochlear and auditory nerve properties in different species and for human psychophysical data. Some models are biologically detailed and accurately capture particular response properties of the auditory nerve (6, 11–16), while others are abstracted approximations of the signal transformation in the auditory periphery (17–19). Some have been used to provide inputs for models of auditory neurons (17–21), to generate perceptual models (22), and in machine processing of sounds (2, 23). However, few attempts (24) have been made to determine which cochlear models best describe the computational impact of the auditory periphery on neural responses in mammalian auditory cortex, although more progress has been made in the avian auditory system (25). The models that best explain particular physiological characteristics of the auditory periphery may differ from the ones that best explain the impact of auditory nerve activity on cortical responses to natural sounds. This is because neuronal responses are transformed through the central auditory pathway to the cortex, and the periphery may operate differently with natural sounds.

Here we considered a range of existing biologically detailed models of the auditory periphery and adapted them to provide input for a number of encoding models of cortical responses. We also constructed a variety of simple spectrogram-based models, including one accounting for the different types of auditory nerve fiber. Surprisingly, we found that the responses of neurons in the primary auditory cortex (A1) in ferrets can be explained equally well using the simple spectrogram-based cochlear models as when more complex biologically detailed cochlear models are used. Furthermore, the simple models explain the cortical responses more consistently well over different sound types and anesthetic states. Hence, much of the complexity present in auditory peripheral processing may not substantially impact cortical responses. This suggests that the intricate complexity of the cochlea and the central auditory pathway together results in a simpler than expected transformation of auditory inputs from ear to cortex.

## Results

### Generating Cochleagrams Using Cochlear Models.

In this study, we consider two broad classes of cochlear models. The first class is based on cochlear filterbanks and has somewhat more detailed biological underpinnings than the second class. We consider several models of this first class, which we refer to here as the Wang Shamma Ru (WSR) model (3–5), the Lyon model (2, 10), the Bruce Erfani Zilany (BEZ) (14, 15, 26) model, and the Meddis Sumner Steadman (MSS) model (6, 7, 11, 13, 16). These models vary substantially in their filterbanks and compression functions (see *SI Appendix*, *Methods* for details). The WSR model has logarithmically spaced filters, followed by nonlinear compression, lateral inhibition, and leaky integration (27). The Lyon model has a near-log spacing of frequency channels that becomes more linear near the low frequencies. The frequency decomposition is accompanied by an adaptive-gain control mechanism that acts as the compression function (2, 10). The BEZ model includes multiple detailed stages of signal transformation to mimic various stages of the processing by the ear and the auditory nerve of the cat (14, 26, 28). The MSS model is similar to the BEZ model in that it also models the processing stages from the ear to the auditory nerve, but of a different species, the guinea pig (6, 7) (see *SI Appendix*, Fig. S1 for schematic diagrams of each cochlear model). Recent work suggests the ferret peripheral auditory system is comparable to that of other mammalian species (29), such as cats and particularly guinea pigs (30).

The second class of models are the STFT (short-time Fourier transform) spectrogram-based models—these models are aimed at approximating the information processing in the auditory periphery without modeling the detailed biological mechanisms (17–19, 31). Implementation of these models consists of three key components: frequency decomposition, response integration, and compression. We constructed the spectrogram-based cochlear models by performing frequency decomposition using an STFT of the sound waveform. The amplitude or power spectrogram was then put through a weighted summation using overlapping triangular filters spaced on a logarithmic scale to obtain specified numbers of frequency channels. Finally, nonlinear compression was applied. For the amplitude spectrogram-based models, the compression functions used were a thresholded log function and a log(1+(⋅)) function. We refer to these models as the spec-log and spec-log1plus, respectively. For the power spectrogram-based models, a thresholded log compression function was used either alone or together with a Hill function; we refer to these models as the spec-power and spec-Hill models (*SI Appendix*, Fig. S1).

**Fig. 1.**
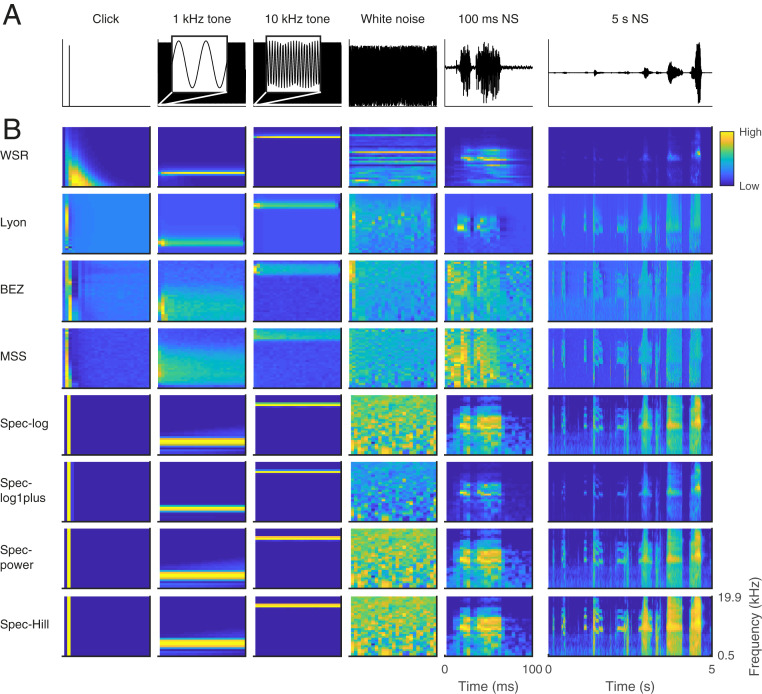
Cochleagram produced by each cochlear model for identical inputs. (*A*) Each column is a different stimulus: a click, 1-kHz pure tone, 10-kHz pure tone, white noise, a natural sound—a 100-ms clip of human speech—and a 5-s clip of the same natural sound (*Left* to *Right*). (*B*) Each row is a different cochlear model.

Each cochlear model produces a characteristic cochleagram for the same sound input. We illustrate this by presenting a range of synthetic and natural sound inputs (Fig. 1*A*) to each model. Fig. 1*B* shows the cochlear models’ responses to a click, pure tones of 1 and 10 kHz, white noise, and natural sounds. Here we depict cochleagrams with 32 frequency channels, although we studied the impact of varying the number of channels in each model, typically examining 2, 4, 8, 16, 32, 64, and 128 frequency channels. For a click input, cochleagrams produced by spectrogram-based models have sound activity tightly localized in time, but cochleagrams produced by filterbank-based models are more temporally spread, with the response persisting after the impulse occurred (Fig. 1*B*). For pure-tone input, cochleagrams produced by all models look similar except for the Lyon, BEZ, and MSS models, where the cochleagram is broader in frequency content than in the other models (Fig. 1*B*). For white noise, most models have responses smoothly distributed across frequency, except for the WSR model.

Cochleagrams of natural sounds also differ between cochlear models (Fig. 1*A* and *SI Appendix*, Fig. S2). However, two filterbank-based models (the BEZ and MSS models) produce similar-looking cochleagrams, as do three spectrogram-based models (spec-log, spec-power, and spec-Hill). Overall, the WSR model produced very different cochleagrams across a range of stimuli (click, white noise, and natural sounds). Furthermore, the maximum energy in the cochleagrams of the spec-log1plus model is lower than other spectrogram-based models. Compared with other models, the output of the BEZ and MSS models looks noisier due to the stochasticity in their models of inner hair cells, ribbon synapse vesicle release, or auditory nerve firing. We therefore averaged across multiple repeated runs of these models to provide cochleagrams that reduced this variability (see *SI Appendix*, *Methods* for more details). We quantified the similarity between the cochleagrams produced by each cochlear model for natural sound inputs by calculating the correlation coefficients between the cochleagrams produced by each possible pair of cochlear models. This quantitative analysis supports our qualitative observations on the similarities and differences between the cochleagrams of the different models (*SI Appendix*, Fig. S3).

### Predicting Responses of Auditory Cortical Neurons Using Different Cochleagram Inputs.

The datasets used in this study were from extracellular recordings of the responses to sounds of neurons in ferret A1. We used three datasets: responses to natural sounds in anesthetized ferrets [natural sound dataset 1; NS1 (32)], responses to dynamic random chords (DRCs) in the same anesthetized ferrets [DRC dataset (32)], and responses to natural sounds in awake ferrets [natural sound dataset 2; NS2 (33)]. We will focus first on the results with NS1, which consisted of neural responses to a diverse selection of natural sounds (20 sound snippets, each 5 s in duration), including human speech, animal vocalizations, and environmental sounds (17–19). This dataset constitutes a total of 73 single units, which were those units with a noise ratio (34, 35) of <40 so as to exclude neurons whose response showed little dependence on the stimulus (see *SI Appendix*, *Methods* and ref. 17 for details).

**Fig. 2.**
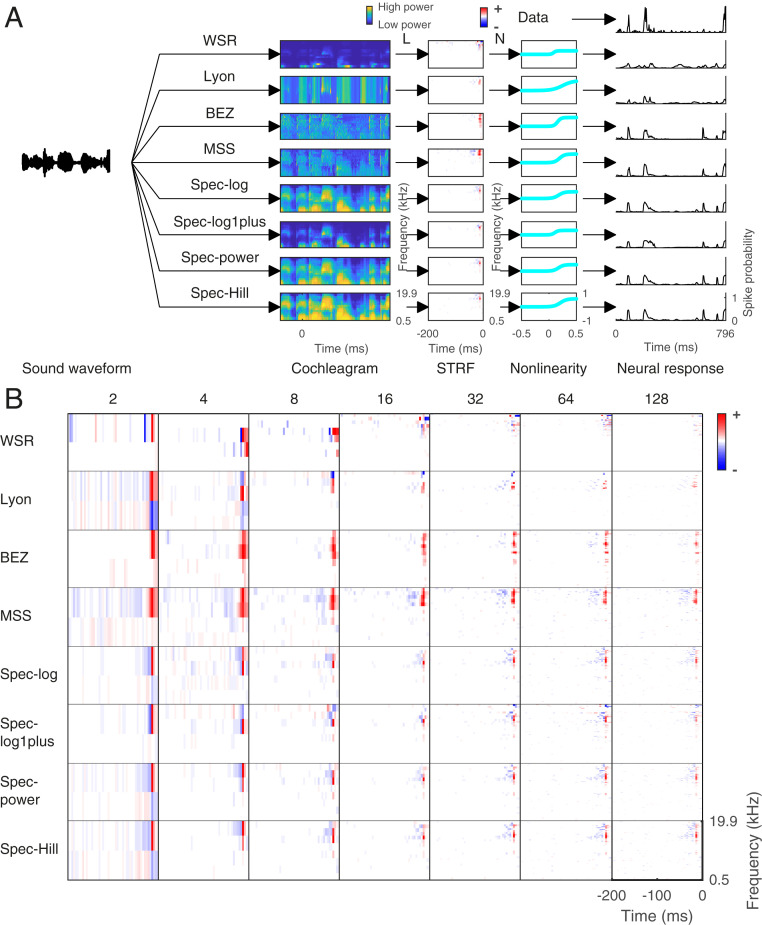
Estimating spectrotemporal receptive fields. (*A*) The encoding scheme: preprocessing by cochlear models to produce a cochleagram (in this case, with 16 frequency channels) followed by the linear–nonlinear encoding model. The parameters of the linear stage (the weight matrix) are commonly referred to as the spectrotemporal receptive field of the neuron. Note how the choice of cochlear model influences estimation of the parameters of both the L and N stages of the encoding scheme and, in turn, prediction of neural responses by the model. (*B*) The STRF of an example neuron from natural sound dataset 1, estimated by using different cochlear models. Each row is for a cochlear model and each column is the number of frequency channels.

The sound pressure waveform is generally not a suitable input to an encoding model of a neuron in A1. A better choice of input is typically a frequency-decomposed version of the sound (20, 25, 34, 36–47) that resembles the peripheral processing in the cochlea. Cochlear models are often used as input to models of responses of auditory cortical neurons (17–19, 25, 35, 48), such as the commonly used linear–nonlinear (LN) model of neural responses (35, 49). Hence, we use a two-stage encoding framework to estimate firing-rate time series in response to natural sounds of neurons in ferret A1. The first stage of the encoding framework processes the sound stimuli using a cochlear model to generate a cochleagram (Fig. 2*A*). The second stage estimates the firing-rate time series as a function of the preceding cochleagram using an LN model (*SI Appendix*, *Methods*). An LN model was fitted individually to each unit’s responses to 16 of the 20 sound snippets using *k*-fold (*k* = 8) cross-validation and L1 regularization (50) (the distribution of the values of the regularization parameter is shown in *SI Appendix*, Fig. S4). Details of the cross-validation procedure and parameter estimation have been described previously (17) (also see *SI Appendix*, *Methods*). We fitted an LN model for each cochlear model with a specified number of frequency channels (2, 4, 8, 16, 32, 64, and 128).

The linear part (L) of the LN model captures the linear dependence of a neuron’s firing rate on the frequency content of the cochleagram at different time delays, namely the spectrotemporal receptive field (STRF) (20, 25, 34, 36–39, 41–47). STRFs are widely used to describe the stimulus feature selectivity of auditory cortical neurons. The general properties of STRFs estimated for the same neuron using different cochlear models were similar (Fig. 2*B*). All cochlear models produced STRFs that contained excitatory and lagging inhibitory fields. The shape of the STRFs produced by different models also resembled each other. The largest weight in the STRF occurred at a comparable frequency (best frequency) and time (latency) for all models and regardless of the number of frequency channels. The only exceptions to this were the 2- and 4-frequency channel models, which sometimes showed very different frequency selectivity, presumably because of the very limited choice of frequency channels (*SI Appendix*, Fig. S5). The ratio of inhibitory vs. excitatory field strength (IE score) (17, 19) was also very similar for STRFs produced by different cochlear models, with the exceptions of the WSR and BEZ models (*SI Appendix*, Fig. S5).

Although the general properties of the STRFs obtained using different cochlear models were similar, a more detailed analysis revealed some variability between pairs of STRFs estimated for the same neuron using two different cochleagram models (*SI Appendix*, Fig. S6 *A* and *B*). Higher correlations were observed between STRFs estimated from the same class of cochlear models. In particular, spec-log, spec-power, and spec-Hill models produced very similar STRFs, whereas this was less true of the spec-log1plus model. STRFs obtained with the MSS and BEZ models were similar to each other and, to a lesser extent, to the spec-Hill model. Applying Gaussian blurring to account for frequency or temporal shifts in the STRFs improves the correlations, but did not change the overall trends in these results (*SI Appendix*, Fig. S6*C*).

**Fig. 3.**
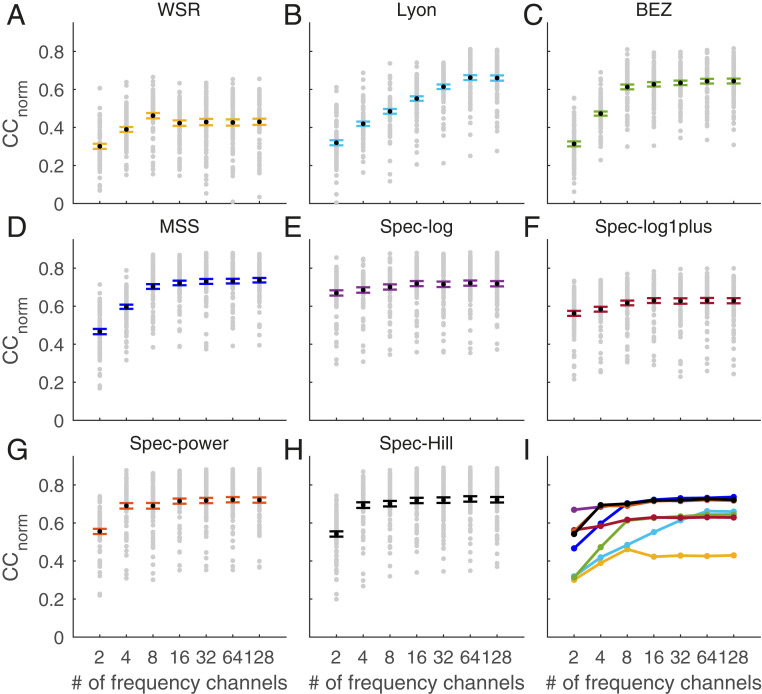
Performance of different cochlear models in predicting neural responses of NS1. (*A*) WSR model. (*B*) Lyon model. (*C*) BEZ model. (*D*) MSS model. (*E*) Spec-log model. (*F*) Spec-log1plus model. (*G*) Spec-power model. (*H*) Spec-Hill model. Each gray dot represents the *CC*_norm_ between a neuron’s recorded response and the prediction by the model; the larger black dot represents the mean value across neurons and the error bars are SEM. (*I*) Comparison of all models. Color coding of the lines matches the other panels.

The models vary in how well they match the peaks in the responses of A1 neurons. Likewise, the measured overall prediction performance of the LN model on a held-out dataset differed between different cochlear models. As a measure of the prediction performance, we used the normalized correlation coefficient (*CC*_norm_) (31) over all neurons in the dataset, where a *CC*_norm_ of 0 indicates no correlation between the neural response and the model’s estimate and a *CC*_norm_ of 1 indicates that the model can predict all variance in the firing rate (averaged over repeats) that depends on the stimulus. We found that the mean *CC*_norm_ over all neurons varied depending on the choice of cochlear model and the number of frequency channels in the cochleagram (Fig. 3 and *SI Appendix*, Table S1). We define the peak *CC*_norm_ of a model as the highest mean *CC*_norm_ across the number of frequency channels. The peak *CC*_norm_ was 0.462 for the WSR model (at 8 channels), 0.662 for the Lyon model (at 64 channels), 0.644 for the BEZ model (at 128 channels), 0.725 for the MSS model (at 128 channels), 0.721 for the spec-log model (at 64 channels), 0.630 for the spec-log1plus model (at 64 channels), 0.722 for the spec-power model (at 64 channels), and 0.726 for the spec-Hill model (at 64 channels) (Fig. 3*I* and *SI Appendix*, Table S1). A log-spaced power spectrogram with successive log and Hill compression functions (spec-Hill) provided the best prediction performance, with a mean *CC*_norm_ of 0.726 (*SI Appendix*, Table S1). However, three of the spectrogram models, the spec-log, spec-Hill, and spec-power, and one of the biological models, MSS, all predicted similarly well at about 0.72 to 0.73 peak *CC*_norm_. In contrast, one of the spectrogram models, spec-log1plus, and three of the biological models, WSR, Lyon, and BEZ, predicted substantially less well, with peak *CC*_norm_ in the range of 0.45 to 0.66 (*SI Appendix*, Table S1).

Selecting the best model for individual neurons supports the findings based on the average performance of each model for all neurons. The spec-Hill and MSS models with either 64 or 128 frequency channels provided the best prediction performance for most neurons (*SI Appendix*, Fig. S7). We also compared the predicted response obtained with the MSS model with the predicted response of the other models and found that the similarity in peak *CC*_norm_ performance generally covaried with the similarity in predicted response (*SI Appendix*, Fig. S8).

### Multifiber Cochleagrams.

We have used the word “cochleagram” so far to refer to a time-frequency representation of the sound stimulus, with a single output for each time and frequency. In the auditory system, however, afferent nerve fibers tuned to the same frequency can have different sound-intensity thresholds and dynamic ranges, and three different auditory nerve fiber types have been physiologically characterized (30, 51, 52). The three types of fibers are low spontaneous rate (LSR) with higher threshold and larger dynamic range, medium spontaneous rate (MSR) with intermediate threshold and dynamic range, and high spontaneous rate (HSR) with lower threshold and narrower dynamic range. To study the impact of this representation on the prediction performance of modeled cortical responses, we used an MSS model with the three different fiber types (multifiber MSS model) as input to the LN model. We also constructed a multithreshold spec-Hill model, where each frequency channel went through three different Hill functions with different thresholds and dynamic ranges (*SI Appendix*, *Methods*). This produced a cochleagram representation that assigns the changing sound level in a single frequency channel to three separate channels, analogous to the three fiber types in the multifiber MSS model.

**Fig. 4.**
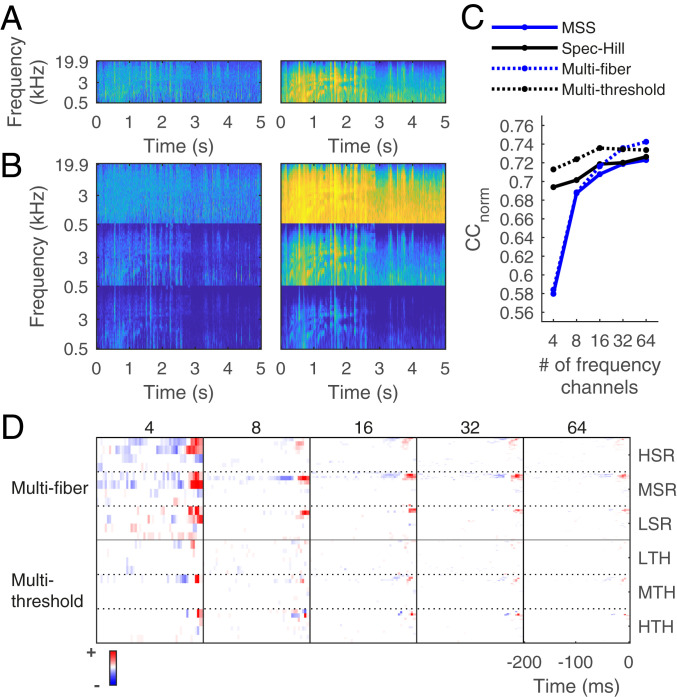
Multifiber and multithreshold cochlear models. (*A*) Cochleagram of a natural sound clip produced by the MSS model (*Left*) and the spec-Hill model (*Right*). (*B*) Cochleagram of the same natural sound clip produced by the multifiber MSS model (*Left*) and the multithreshold spec-Hill model (*Right*). (*C*) Mean *CC*_norm_ for predicting the responses of all 73 cortical neurons in NS1 for the multifiber/threshold models and their single-fiber/threshold equivalents. (*D*) STRFs of an example neuron from NS1, when estimated using the multifiber and multithreshold models. HSR, high spontaneous rate; MSR, medium spontaneous rate; LSR, low spontaneous rate; LTH, low threshold; MTH, medium threshold; HTH, high threshold.

When we used these models as input to the LN model of cortical neurons, we were able to predict cortical responses to natural sounds slightly better than the single-fiber or single-threshold versions of the models (Fig. 4). For the NS1 dataset, the multithreshold spec-Hill model performed better than the multifiber MSS model for cochleagram inputs with fewer than 32 center frequencies but performed slightly worse for cochleagram inputs with 32 or more center frequencies (Fig. 4). Detailed values of mean *CC*_norm_ are given in *SI Appendix*, Table S1.

### Generality of the Model Performance and Further Explorations.

**Fig. 5.**
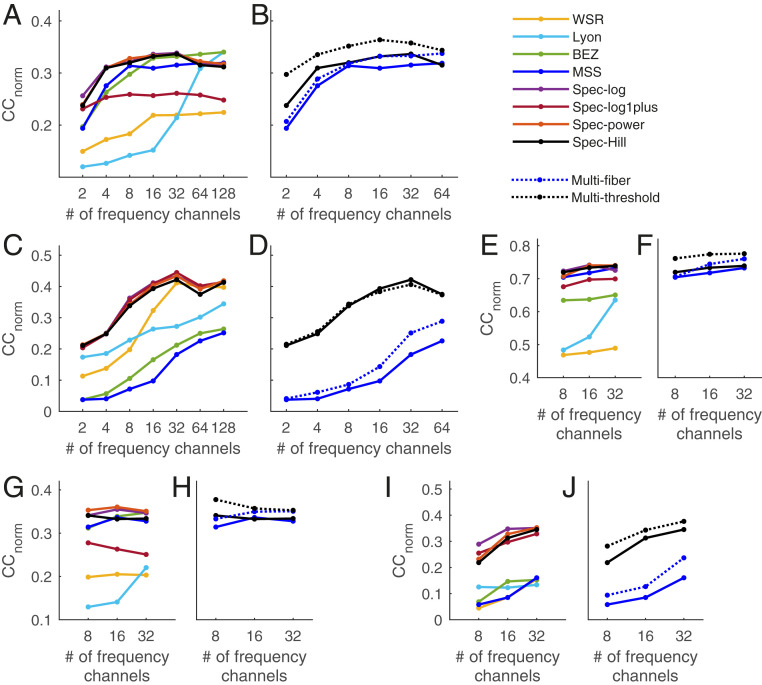
Performance of different cochlear models across datasets and encoding models. (*A* and *B*) Mean *CC*_norm_ between the LN encoding model prediction and actual data for all neurons in natural sound dataset 2 (awake ferrets) for single-fiber models (*A*) and for multifiber models (*B*). (*C* and *D*) Mean *CC*_norm_ between the LN encoding model prediction and actual data for all neurons in the DRC dataset (anesthetized ferrets) for single-fiber models (*C*) and for multifiber models (*D*). (*E* and *F*) Mean *CC*_norm_ between the prediction of the NRF model and actual data for all neurons in NS1 (anesthetized ferrets) for single-fiber models (*E*) and for multifiber models (*F*). (*G* and *H*) Mean *CC*_norm_ between the prediction of the NRF model and actual data for all neurons in NS2 for single-fiber models (*G*) and for multifiber models (*H*). (*I* and *J*) Mean *CC*_norm_ between the prediction of the NRF model and actual data for all neurons in the DRC dataset for single-fiber models (*I*) and for multifiber models (*J*).

While we aimed with natural sound dataset 1 to have a diverse and representative stimulus, this does not, of course, represent the full space of natural sounds. Moreover, our electrophysiology data came from anesthetized animals, raising a question over whether brain state might affect model performance. To examine how the choice of stimulus and brain state influences the results, we tested the performance of the models on two other datasets. One of these (NS2) consisted of extracellular recordings from A1 of awake ferrets in response to a different set of natural sounds (18 sound snippets, each 4 s in duration), including human speech, animal vocalizations, music, and environmental sounds (53). In total, 235 single units were included, which had a noise ratio of <40. Using the same methods as for NS1, we used this new dataset to train and test the models’ performance (*SI Appendix*, *Methods*). For this dataset, *CC*_norm_ values were lower for all models (Fig. 5 *A* and *B* and *SI Appendix*, Table S1). Considering the peak *CC*_norm_ values, we found that the same simple spectrogram-based models (spec-log/power/Hill) remained among the top-performing models, performing similar to the best biological models (Lyon/BEZ) (Fig. 5*A*), at 0.33 to 0.34 peak *CC*_norm_. However, the best-performing biological models were not the same as for NS1, with the MSS model now performing poorly compared with the Lyon and BEZ models (*SI Appendix*, Table S1). These worse-performing models (spec-log1plus, MSS, and WSR) had peak *CC*_norm_ values within the range of 0.22 to 0.32 (*SI Appendix*, Table S1).

We also tested the performance of the models on a different type of stimulus. The 73 neurons in NS1 were also played 12 DRC stimuli, which consisted of randomly constructed chords changing every 25 ms. Using the same methods as for NS1, we used this DRC dataset to train and test the models’ performances (*SI Appendix*, *Methods*). We found that the same simple spectrogram-based models (spec-log/power/Hill) remained among the top-performing models, now joined by the spec-log1plus model (Fig. 5 *C* and *D* and *SI Appendix*, Table S1). They performed slightly better than the best biological model (WSR) (Fig. 5*C* and *SI Appendix*, Table S1), with peak *CC*_norm_ in the range of 0.42 to 0.45 compared with the 0.41 peak *CC*_norm_ of the WSR model. However, the biological models changed in which ones performed best, now with the MSS model (best for NS1) and Lyon/BEZ models (best for NS2) no longer being comparable to the spectrogram models, and instead the WSR model resembling the performance of the simpler spectrogram models. These worse-performing models (MSS, BEZ, and Lyon) had peak *CC*_norm_ values in the range of 0.25 to 0.34 (Fig. 5*C* and *SI Appendix*, Table S1). Thus, while the spec-log/Hill/power models show consistently high performances for all three datasets, the performance of the other more biological models varies substantially from dataset to dataset.

We found that for the NS2 and DRC datasets the multithreshold model outperformed the multifiber model. For the NS2 dataset, we also found that both the multifiber model and multithreshold model performed better than their single-fiber/threshold equivalent models. However, for the DRC dataset, while the multifiber model performs better than its single-fiber equivalent (MSS) (Fig. 5*B*), the multithreshold model does not perform better than its single-threshold equivalent (spec-Hill) (Fig. 5*D*). Detailed values of the prediction performance metric for all models and these two additional datasets are given in *SI Appendix*, Table S1.

So far, we have reported the prediction performance of the cochlear models when combined with an LN encoding model. To what extent does the choice of encoding model influence the results? We tested the prediction performance of just the linear stage (the STRF) of the LN model and found that *CC*_norm_ values are lower than those for the LN model. However, the performance of the models remains largely unchanged in comparison with one another (*SI Appendix*, Fig. S9). Furthermore, we tested the performance of each cochlear model when combined with a network receptive field (NRF) encoding model (see *SI Appendix*, *Methods* for more details) (17, 19). This is a single hidden layer neural network with units with sigmoid nonlinear activation functions. The NRF model has a higher number of parameters and is hence likely more sensitive to the amount of training data than the LN model. To keep the parameter number low and to save the running time, we ran these models with a limited set of frequency channel numbers. The *CC*_norm_ values for the NRF model for both natural sound datasets are typically higher than for the LN model. The NRF model values for natural sounds are particularly high when the NRF model is combined with the multithreshold model (Fig. 5 *F*, *H*, and *I* and *SI Appendix*, Table S2), reaching up to 0.78 for NS1. However, the relative performance of different cochlear models remains similar to the LN model (Fig. 5 *E*–*J* and *SI Appendix*, Table S2).

For all datasets, we examined the consequence of using *CC*_norm_, by examining how the results looked for a different commonly used measure, the raw correlation coefficient. The *CC* varies across the different models in a very similar way to the *CC*_norm_ (*SI Appendix*, Fig. S10). We also explored in more detail the consequences of some of our other modeling choices, this time just for NS1. First, there is a stochastic element to the MSS and BEZ models. We investigated the effect of this noise in the MSS and BEZ models on the prediction performance. For predicting cortical responses, we used the MSS and BEZ response averaged over 20 repeats to lessen the stochasticity. When we take the average of 100 or 200 repeats, the *CC*_norm_ of the MSS model is very similar to the MSS with 20 repeats, indicating that averaging over 20 repeats is sufficient (*SI Appendix*, Fig. S11). The effect of repeats is also similar for the BEZ model (*SI Appendix*, Fig. S12). Second, for model training and testing, we initially excluded onset responses from the neural data (the first 800 ms), as is common practice in STRF estimation (18, 19, 54). However, we have examined the consequence of including the onset responses, and we found that including them has very little effect on the performance of the LN models, regardless of the cochlear model used for preprocessing (*SI Appendix*, Fig. S13).

We also explored what nonlinear aspects of the spectrogram cochlear model and LN model combination are important for good prediction of cortical neural responses. Both the cochlear model and the LN encoding model include nonlinearities. To examine how these two nonlinearities interact, we constructed a spectrogram cochlear model without any compressive cochlear nonlinearity (spec-lin) and compared its performance with the other spectrogram models that included a compressive cochlear nonlinearity, in the presence or absence of the LN model output nonlinearity. Although there is some variation across nonlinearities and stimulus sets, in general we found that the compressive cochlear nonlinearity and the output nonlinearity contributed to prediction partially independently. When a model had both nonlinearities together, it typically predicted better than just one nonlinearity on its own, but a compressive cochlear nonlinearity tended to contribute more than the output nonlinearity (*SI Appendix*, Fig. S14).

Finally, we have extended our analysis beyond the average *CC*_norm_ for a whole dataset, by exploring how the predictive capacity of the model fits depends on different features of the neurons or stimuli. To display the relative performance of individual neurons, scatterplots of the *CC*_norm_ of every neuron for each model, plotted against the MSS model, are given for all three datasets in *SI Appendix*, Fig. S15. Neurons vary in their noise ratio and, for the natural sounds, *CC*_norm_ showed little dependence on noise ratio, whereas for the DRC stimuli, noisy neurons tended to have lower *CC*_norm_ values (*SI Appendix*, Fig. S16). We also examined how *CC*_norm_ depended on the neuron’s best frequency and IE score (17, 19). No strong relationships were apparent (*SI Appendix*, Figs. S17 and S18). When we examined the dependence of *CC*_norm_ on latency, it did appear that longer-latency neurons had lower *CC*_norm_ values (*SI Appendix*, Fig. S19). This is consistent with them perhaps having additional nonlinearities due to receiving stronger inputs from higher cortical areas, as suggested by their long latency. We also explored how well neural responses were predicted for the four stimulus types in the test set of NS1: ferret vocalizations, other animal sounds, speech, and environmental sounds. The spec-log/power/Hill models and the MSS model remained consistently among the top-performing models for each stimulus type, indicating that the robustness of the spec-log/power/Hill models is not driven by a subset of stimuli (*SI Appendix*, Fig. S20). Finally, to explore which aspects of the neural response are better predicted by the different cochlear models, we examined how the mean squared error (MSE) of the model’s estimate of the cortical response depended on the recorded spike probability (*SI Appendix*, Fig. S21). We found that models that performed well tended to have lower MSE during the high spike probability times as compared with models that performed less well, suggesting that predicting peaks in high spike activity accurately is a factor in determining model performance.

## Discussion

In this study, we aimed to uncover the computational transformation of the auditory signal from the ear to the cortex. To do this, we investigated and developed different models of the auditory periphery, and assessed their capacity to provide the input to encoding models of the responses of auditory cortical neurons to a range of sounds, including natural sounds. Surprisingly, we found that the only models that consistently predicted the responses of A1 neurons across datasets, stimulus type, and brain state were the simple models that were based on little more than a spectrogram and some compression (the models spec-log, spec-power, and spec-Hill, which all performed similarly). Likewise, a simple spectrogram-based model that approximated the three fiber types of the auditory nerve (the multithreshold model) tended to produce better predictions of the neural responses than a complex model with extensive biological detail and the three fiber types. These findings hold when the models were used as input to different encoding models [linear, linear–nonlinear, and network receptive fields (17, 19)], emphasizing their robustness. These findings suggest that the functional transformation from the ear to the auditory cortex might be simpler than expected and that many of the details of the mechanical and neural properties of the ear, and the tuning properties of the auditory nerve and brainstem, may be of limited relevance to their impact on cortex. This exemplifies the distinctions made by Marr and Poggio (1) between the computational and algorithmic levels of analysis, which in this case may be surprisingly simple, and the implementation level, which is very complex.

The observed changes in encoding model prediction performance with different cochlear models were substantial in size, similar to those resulting from inclusion of features such as gain control (35) and network structure (17, 19) in encoding models of cortical neurons. The choice of cochlear model is therefore likely to be an important factor in accounting for differences in prediction performance of similar encoding models reported by different groups (18, 20, 48). Furthermore, the addition of multiple fiber types/thresholds can produce improvements in the performance of the model. Additional improvements occur when multiple fiber types/thresholds were used in conjunction with network structure to predict the responses of NS1, with prediction performance reaching a remarkably high *CC*_norm_ of 0.78 (highest achieved so far for this dataset, compared with refs. 17–19) for the multithreshold model. Nonlinear features often do not improve prediction independently. However, the NRF and multithreshold nonlinearities appear to be relatively independent, both contributing to prediction when applied together. Similarly, with the single-threshold spectrogram models, the cochlear model compression acts relatively independent of the LN model output nonlinearity.

One reason why the spectrogram models performed both well and consistently across stimulus types may be that the biological models fail to accurately represent the processing that takes place in the mammalian auditory periphery generally or the ferret auditory periphery specifically. The WSR and Lyon models are based on the broad results of animal experiments and designed to match certain human psychophysical percepts. The MSS and BEZ models are derived from detailed guinea pig data and cat data, respectively (6, 7, 11, 12), and verified with auditory nerve responses to simple stimuli. Although there are relatively few physiological studies of the ferret peripheral auditory system, estimates of cochlear frequency selectivity in ferrets (29, 30) are comparable to those made for other mammalian species, notably guinea pigs and cats. Recent work suggests that frequency selectivity of ferrets more closely resembles that of guinea pigs than cats (30). Hence, one might expect the MSS model to perform best, but this is only the case for NS1, not NS2 or the DRC dataset. The biological models will have had their many complexities adjusted to account for physiological or psychophysical phenomena based on simple stimuli. Hence, it may be that each model only predicts cortical responses well for stimuli containing features around which the model was constructed. Our simple spectrogram models, in contrast, are not specialized for capturing particular stimulus features. This perhaps renders the simple spectrogram models more representative of the transformation performed by the periphery across a broad span of sounds, including natural sounds.

Another possible reason why the spectrogram models perform better than the biological models has to do with the fact that the input to the cortex does not come directly from the auditory nerve. Considerable processing takes place along the auditory pathway, with neurons at each stage being increasingly low-pass to fine-structure (55–57) and amplitude modulation (58) and becoming invariant to various features of the stimulus such as acoustic background noise (59). This results in cortical responses being relatively insensitive to the fine temporal structure and fast amplitude modulation of sounds that are precisely encoded by auditory nerve fibers (55–58). Furthermore, stimulus features can be transformed nonlinearly into quite different representations at higher levels of the auditory pathway (58). This processing may explain why the resulting transformation from ear to cortex is better captured by our simpler spectrogram models than by the cochlear models with more extensive detail of auditory nerve responses.

It is important to consider a few caveats for our results. The dependence of model predictions on stimulus set (18, 20, 60) and encoding model (24, 61) is well-recognized. We have shown the robustness of our simple cochlear models over three datasets that differ in stimulus type (natural sounds and DRCs) and brain state (awake and anesthetized), and using three different encoding models (L, LN, and NRF). However, other factors, such as spatial hearing cues, were not included, and reverberation, background noise, and sound mixtures were only present to a limited extent. Furthermore, a proportion of the stimulus-dependent neural response in A1 could not be explained by any of the models. This is particularly the case for NS2, perhaps due to increased nonlinearity in the awake nervous system (62), and for the dynamic random chords, perhaps due to more spectral detail. This all implies that while our simple models capture much of the transformation from ear to cortex, a more accurate approximation of the transformation, and one that applies more widely, may be more complex. Investigating which aspects of auditory processing at subcortical levels of the auditory pathway are relevant to models of cortical neurons can be determined empirically by similar methods. For example, our results suggest that the division of the auditory signal among the three physiologically distinct categories of auditory nerve fiber is an important detail for the ear-to-cortex transformation.

Our study is an extensive comparison focused on the capacity of different peripheral models to capture cortical neural response in mammals. In a pioneering earlier study in birds, Gill et al. (25) examined how well different cochlear models predicted neural responses to conspecific birdsong and modulation-limited noise in the avian midbrain and the primary and secondary auditory forebrain, which are considered to be the avian homolog of mammalian A1 (63). They found that time-frequency scale and whether logarithmic or linear frequency spacing of filters was used had limited impact, and that the optimal values in the models were stimulus-dependent. The time-frequency scale relates to the number of frequency channels (although it also relates to time resolution, complicating matters). Over an equivalent range of frequency channels (about 20 to 120), we similarly found that the number of channels often had limited impact on prediction and that the optimal number depended on the stimulus and model. However, below ∼20 channels, we generally found more channels to be better. As in our study, Gill et al. (25) also found that sublogarithmic compression, linear and log(1+(⋅)) in our case, linear and power law in theirs, fitted neural responses worse than logarithmic. Similarly, in an investigation of linear encoding models of mammalian A1 neurons (24), which also explored channel number, the peripheral model selected used near-logarithmic compression and 18 channels. This is consistent with our study and that of Gill et al. (25) in showing that a model incorporating logarithmic compression and at least ∼20 frequency channels predicts cortical responses well.

Finally, in contrast to our study, Gill et al. (25) found that Lyon’s model with adaptive gain control provided the best fit to neural responses for both their stimulus types. Reasons for this difference could be the species or stimulus sets used, or details of our spectrogram models such as the use of triangular filters. Gain control and other adaptive phenomena are ubiquitous features of mammalian auditory processing (35, 37, 64–67), so the Lyon model’s underlying transmission-line cochlear model or the parameters of its adaptive-gain control may simply not match well with the adaptation and other features exhibited by the ferret auditory system. An appropriate cochlear model with species-specific gain adaptation may further improve on our results, particularly as adding sound-level adaptation to our spectrogram-based models improves prediction of ferret cortical responses (18).

Relevant to our multithreshold model is a model of A1 responses that also uses multiple level-dependent input nonlinearities (68). This input nonlinearity model transformed the sound levels in each frequency band using a set of fixed basis functions. The basis functions are not biologically inspired, in contrast to our multithreshold model, and were applied to DRCs with 10 discrete sound levels, rather than natural sounds and continuous-valued DRCs. This model predicted rodent A1 responses to DRCs better than an STRF model, but it was not compared with an LN model and the interaction of the model’s input nonlinearity with an output nonlinearity was not explored. Finally, single hidden layer artificial neural networks have been applied to predict ferret A1 responses (19). This network model also has features resembling multiple input nonlinearities (the hidden unit nonlinearities), albeit with a linear transform applied first, and this was shown to predict better than an LN model. However, as we have seen in Fig. 5, appending this network model to our multithreshold model even further improves prediction of A1 responses to natural sounds, indicating that the multiple thresholds and the hidden unit nonlinearities capture different nonlinear aspects of the ear-to-cortex signal transformation.

It is interesting to speculate on the perceptual, behavioral, and clinical implications of our findings. Algorithms for automatically estimating speech quality (and other speech characteristics) are useful for assessing hearing aids and other auditory prosthetics and can also indicate what sound transformations guide perception and action. Wirtzfeld et al. (69) found that a complex biologically detailed cochlear model (15, 26) did not outperform a simpler model (70) in predicting human estimates of speech quality in noise, although the relative effectiveness of different models in assessing speaker identity is affected by the presence of different types of background noise (71). Similarly, a simple cochlear model is sufficient to reproduce the task-dependent STRF plasticity that characterizes the auditory cortex (72). If cortical activity reflects perception and guides behavior, these studies are consistent with our finding that a simple transformation predicts cortical responses well, and also suggests possible value in using our simple models in speech assessment and recognition algorithms. An alternative way to investigate what aspects of cochlear models are important for perception would be to synthesize sound textures (22) using different cochlear models and quantitatively assess human judgments of their quality. Our findings also have implications for cochlear implants, where electrical pulses are delivered directly to the auditory nerve, and particularly brainstem, midbrain, or cortical implants (73), by suggesting simple signal-processing strategies to mimic the impact of the auditory periphery on the stimulated neurons.

In summary, although extensive processing takes place in the cochlea and the central auditory pathway (74), our results suggest that the cortex receives a relatively simple functional transformation of sound inputs. Many of the complex properties of peripheral auditory processing appear to have limited impact on cortical responses, and much of that processing is captured by a simple spectral decomposition of the input. Explaining the remaining aspects of how cortical neurons respond to natural sounds will likely require additional complexities to those found in the models we examined, which can be revealed using empirical computational methods such as those adopted here. It is likely that similar principles apply to other sensory systems.

## Methods

Neural responses to sound stimuli were recorded from ferret primary auditory cortex. The sounds were put through a cochlear model which then provided input to an encoding model. The parameters of the encoding model were optimized to estimate the time course of the neural responses to the sounds, and the model was then tested on how well it could predict responses to sounds that were not used for the optimization. The average capacity of different cochlear models to predict the neural responses via encoding models was examined, to determine which cochlear model best captured the impact of cochlea processing on the neural responses (see *SI Appendix*, *Methods* for detailed methodology).

All data obtained by the authors were from experiments performed under license from the UK Home Office and approved by the University of Oxford Committee on Animal Care and Ethical Review.

## Supplementary Material

Supplementary File

Supplementary File

Supplementary File

Supplementary File

## Data Availability

All study data are included in the article and *SI Appendix*. All codes are available at https://github.com/monzilur/cochlear_models.
