# Supplementary material for: Simple transformations capture auditory input to cortex
Source: Proc Natl Acad Sci U S A. 2020 Oct 23;117(45):28442–51. doi: 10.1073/pnas.1922033117 (PMC7668077; doi:10.1073/pnas.1922033117)
Supplement: Supplementary File [file pnas.1922033117.sapp.pdf]

PNAS
www.pnas.org

Supplementary Information for

Simple transformations capture auditory input to cortex

Monzilur Rahman, Ben D. B. Willmore, Andrew J. King, Nicol S. Harper

* Monzilur Rahman, Nicol S. Harper

Email: monzilur.rahman@dpag.ox.ac.uk, nicol.harper@dpag.ox.ac.uk

This PDF file includes:

Supplementary text

Figures S1 to S21

Tables S1 to S2

SI References

Legends for Audio Files S1 to S3

Other supplementary materials for this manuscript include the following:

Audio Files S1 to S3

www.pnas.org/cgi/doi/10.1073/pnas.1922033117

Supplementary Information Text

Supplementary Methods

Codes availability

All codes are available at: https://github.com/monzilur/cochlear_models.

The dataset: stimuli and neural response

Three datasets were used in this study: natural sound dataset 1 (NS1), natural sound dataset 2 (NS2), and the dynamic random chord (DRC) dataset. Each dataset consisted of a number of sound clips, and the neural responses to these clips.

Natural sound dataset 1 (NS1)

This dataset was gathered by the authors and used in previous studies, in some cases as the sole dataset in a study (1, 2) and others as part of the dataset [experiment 1 in (3); part of the "big nat" dataset in (4)]. The dataset is publicly available at https://osf.io/ayw2p/. All data were obtained from experiments performed under license from the UK Home Office and approved by the University of Oxford Committee on Animal Care and Ethical Review. They were obtained using 16- or 32-channel silicon probe electrodes (Neuronexus Technologies) from anesthetized ferrets (ketamine, 5 mg/kg/h; medetomidine, 0.022 mg/kg/h). *In vivo* extracellular recordings were made from the primary auditory cortical areas (primary auditory cortex, A1 and anterior auditory field, AAF, which we collectively refer to as A1) in response to natural stimuli. The sounds were presented binaurally through earphones (Panasonic RPHV297) attached to otoscope speculae inserted into the ear canals, using a sampling rate of 48828.125 Hz. The sound stimuli consisted of 20 clips of natural sound (speech, ferret vocalizations, other animal vocalizations, and environmental sounds), each 5 s in duration. The clips were played in random order. Each clip was repeated 20 times. The RMS sound intensity over the full 5 s duration of each clip ranged from 75 to 82 dB SPL. The RMS sound intensity within 4 ms time bins varied from 29 dB SPL to 105 dB SPL over the whole dataset. In total, the dataset included 549 single and multi-unit recordings from six adult pigmented ferrets (five female and one male), among which 284

were single units. Seventy-three of the single units had noise ratios (5, 6) to the stimulus set of < 40, and these were the units used in this study.

Natural sound dataset 2 (NS2)

The second natural sound dataset has been published by other authors (7) and is publicly available (https://zenodo.org/record/3445557#.XyNEy-d7lEY). A description of the experimental setting and stimuli can be found in (7). Briefly, extracellular neurophysiological recordings were obtained from (A1 and AAF) of six awake ferrets using 1–4 independently positioned tungsten microelectrodes while the animals were passively listening. Sound was delivered through free-field speakers (Manger W05) positioned at ±30° azimuth and 80 cm distant from the animal, at a sampling rate of 44.1 kHz. The sound stimuli consisted of diverse clips of natural sounds. Most clips were presented only once, but some were repeated a number of times, which varied over experiments. In a subset of experiments, there were 18 natural sound stimuli clips (each 4 s in duration) that were repeated 10 (or sometimes 20) times. We selected this subset of experiments and used the neural responses to these 18 natural sounds for our analysis, as a sufficient of repeats is required for the calculation of noise ratio and the PSTH, and a sufficient number of sound clips is needed for model fitting. This set of 18 natural sound clips included human speech, ferret and other species' vocalizations, natural environmental sounds, and sounds from the animals' laboratory environment. The RMS sound intensity of the full 4 s duration of each clip was 54 dB SPL. The RMS sound intensity within 4 ms time bins varied from -16 dB SPL to 76 dB SPL over the whole dataset. The neural dataset for these 18 stimuli consisted of 336 single units. We used the 235 of these units, selected because they had a noise ratio <40 for the 18 natural sound stimuli.

The DRC dataset

The dynamic random chord (DRC) dataset comes from the same anesthetized ferret experiments as the natural sound dataset 1. The DRC dataset has not previously been analyzed in publications, but is publicly available at https://osf.io/ayw2p/. The DRC stimulus clips were randomly interleaved between the clips in natural sound dataset 1, hence the units in the DRC dataset are the same 73 single units

as in that natural sound dataset. The sound stimuli in the DRC dataset consisted of 12 sound clips each of 5 s duration, and each clip was presented 5 times. Each clip was of dynamic random chords (4, 5, 8, 9), which consisted of a sequence of complex tones, each tone being 25 ms long, presented with no gap between the tones. Each complex tone was composed of 34 superposed pure tones, each of whose levels were independently and randomly chosen. The frequencies of the 34 pure tones were log spaced over 5.5 octaves, from 500 Hz to 22627 Hz, with 1/6 octave spacing. The tone levels were picked from a uniform distribution of spanning either 10, 20, 30 and 40 dB, with a mean of 44 dB SPL. The RMS intensity over the full 5 s duration of each clip ranged from 79 to 88 dB SPL. The RMS sound intensity within 4 ms time bins clips varied from 71 dB SPL to 93 dB SPL over the whole dataset.

Cochlear models

Figure S1 illustrates the similarities and differences between of the cochlear models that we used. Below we describe these models.

WSR model: This model was originally proposed by Wang and Shamma and was later described by Powen Ru (Matlab codes that we are using were adapted from https://github.com/tel/NSLtools) (10–13). We refer to it as the WSR (Wang Shamma Ru) model, after the names of the developers. It makes use of a series of filters, whose center frequencies were spaced logarithmically, followed by a sigmoid compression of the filter outputs. The sigmoid compressed outputs were then passed through a lowpass filtering stage, a lateral inhibition stage (between neighboring frequency channels), and a temporal integration stage. The source of these parameters is not strictly physiological, rather, they are abstracted from animal experiments to match perceptual processing. The filterbank of this model comprised a set of 129 given frequency channels. To make this model comparable to other models, appropriately spaced and sized subsets of the frequency channels were selected.

Lyon model: This model uses a cascade of filters with half-wave rectification and adaptive gain control whose center frequencies are spaced according to a logarithmic scale, except for low frequencies, where the spacing is linear, to simulate the behavior of the cochlea (14, 15). The parameters of the

filters have been largely obtained from human psychophysics experiments. We adapted this model from: https://github.com/google/carfac. This implementation has several free parameters, allowing for flexible choice of spacing and bandwidth of each frequency channel in the filterbank – we set these parameters to values that would generate the desired number of frequency channels between 0.5 Hz and 20 kHz.

BEZ model: This is a phenomenological model of cat auditory nerve fibers (16–18), which we refer to as the BEZ (Bruce Erfani Zilani) model [the model described in (16)], named after its developers (we adapted this model from: https://www.urmc.rochester.edu/MediaLibraries/URMCMedia/labs/carney-lab/codes/UR_EAR_v2_1.zip). This model has various processing stages to match the processing stages in the cochlea, the synapses with the auditory nerve and the spiking properties of the auditory nerve. Sound input is first processed through a middle ear filter, followed by a bank of filters with various complexities and non-linearities to account for inner hair cell and outer hair cell properties, the activity of the hair cells is then processed through a nonlinear model of synaptic neurotransmitter release, uptake of neurotransmitter and spiking of the auditory nerve. Because the spiking is a stochastic process, for each center frequency, each of three auditory nerve fiber types was allowed to spike many times (200 for plotting cochleagrams, and 20 for predicting neural responses to reduce running time) and the average was taken over these trials. After that, we took an average over different fiber types weighted by the ratio of each fiber type in the nerve fiber population.

MSS model and multi-fiber model: Different components of this model have been incorporated and refined over the years; we call it the MSS (Meddis Sumner Steadman) model after the name of its developers (we adapted this model from: https://zenodo.org/record/1345757#.XW4XtXt7lPY). The MSS model is a biologically-detailed model of inner hair cells in the cochlea and the auditory nerve (19–24), the parameters of which are based on measurements from the guinea pig (23, 24). The MSS model includes several stages of processing. The first stage – an outer and middle ear (OME) model – uses a series of linear filter approximation to mimic stapes motion at the oval window. The second stage, the frequency decomposition stage, uses a dual resonance non-linear (DRNL) filter architecture to model the properties of the basilar membrane. A DRNL filter consists of two parallel pathways comprising a

series of bandpass and lowpass filters, one with a nonlinear compression and the other without a nonlinear compression. The outputs of each parallel pathway are then combined to find the velocity of the basilar membrane displacement. Finally, transduction by the inner hair cells is modelled using a differential equation for their membrane potential, the synapse is modelled using probabilistic release of neurotransmitter and the activity of the auditory nerve (AN) fibers involves refractoriness, with the spiking of the AN fibers providing the output (Supplementary Fig. 1.1A-C). We set the center frequency of the non-linear filterbank to be log spaced between 508 Hz and 19,912 Hz. Like the BEZ model, for each center frequency each of three auditory nerve fiber types was allowed to spike many times (200 for plotting cochleagrams, and 20 for predicting neural responses to reduce running time) and the average was taken over these trials. For the main MSS model, the output was averaged over the three fiber types, whereas for the multi-fiber MSS model this was not done. Finally, the output was down-sampled into 4-ms time-bins to find a windowed time-frequency representation.

Spec-log model: A spectrogram was produced from the sound waveform by taking the amplitude spectrum using 8-ms Hanning windows, overlapping by 4 ms. The amplitude of adjacent frequency channels was summed using overlapping triangular windows (using code adapted from melbank.m, http://www.ee.ic.ac.uk/hp/staff/dmb/voicebox/voicebox.html), with log-spaced center frequencies ranging from 508 Hz to 19,912 Hz. The amplitude in each time-frequency bin was converted to log values (using 20 * log(·)) and any value below a threshold was set to that threshold (threshold is -40 for NS1 and DRC, -50 for NS2, set by cross-validation).

Spec-log1plus model: This model is the same as the spec-log model but uses log(1+(·)) as the compression function instead of log(·).

Spec-power model: This model too is the same as the spec-log model, but takes the square of the spectrogram, resulting in a power spectrogram instead of an amplitude spectrogram. This model uses a logarithmic compression function (10 * log(·)).

Spec-Hill model: This model is an extension of the spec-power model where the output of spec-power model is further compressed using a non-linear Hill function (25). Before the spec-power output was put into the Hill function, the magnitude of the spec-power model's threshold was added to this output to ensure that it was non-negative. The Hill function is given by,

$$f(\cdot) = \frac{(c*(\cdot))^{1.77}}{\theta + (c*(\cdot))^{1.77}} \ldots (1)$$

Here, *c* is the scaling factor, which was set to 0.01 and θ is the saturation parameter of the Hill function, which was set to 0.16. These values of c and θ were chosen by cross-validation on the validation set for natural sound dataset 1, and then left unchanged for the other datasets (see below).

Multi-threshold model: The multi-threshold version of the spec-Hill model was constructed by taking the output of log compression in a spec-power model but applying three different thresholds of -40, -30 and -15 and thresholding it using values of 0 10 and 25 dB (26), and then compressing it with a Hill function (Equation 1) with different saturation levels (saturation was achieved by using Equation 1 with various θ values: $3\text{x}10^{-5}$, $5\text{x}10^{-5}$, $8\text{x}10^{-5}$ and c = $1\text{x}10^{-4}$). The threshold, θ and, *c* values were chosen to be comparable to biological measurements, and then θ and *c* were slightly adjusted by cross-validation for natural sound dataset 1. These values were then left unchanged for the other datasets (see below).

The PSTH and normalized cochleagram

For each neural unit in a dataset and for each sound stimulus clip *n*, a peri-stimulus time histogram (PSTH) was made. To construct the PSTH the number of spikes was counted in consecutive 4-ms time bins over the course of the clip and averaged over stimulus repeats. The PSTH to stimulus *n* is henceforth denoted as $y_n[\text{t}]$, where *t* indicates the time bin and goes from *t* = 1 to *T*. During the beginning of a clip presentation the neuron is adapting from silence, which can be a challenge to model. It is therefore common practice in fitting STRF and LN models to not use this period of sound (2, 4, 27). Hence, the first 800 ms of the neural response for each clip was clipped from the data for natural sound dataset 1 (although including the first 800ms made very little difference to the results, see Figure S12).

This clipping was not done for natural sound dataset 2 or the DRC dataset as these clips were shorter than those of natural sound dataset 1 and this clipping would have shortened them too much.

The output of each cochlear model is called a cochleagram: the frequency-decomposed transformation of sound over specific time windows (4 ms in our case). To provide input for an encoding model of an auditory cortical neuron, the cochleagrams were normalized to zero mean and unit variance and for each snippet *n*, for every time *t*, a time-lagged matrix was extracted from the cochleagram. This was done according to the equation, $z_{fqn}[t] = k_{fn}[t - q + 1]$, where *k* is the input vector, *f* is the frequency channel, which goes from *f* = 1 to *F*, *q* is the time lag, which goes from *q* = 1 to the maximum time lag Q, and $k_f[t]$ is the cochleagram. The resulting tensor $z_{fqn}[t]$ is the input to the linear nonlinear (LN) stage of the encoding scheme. Here, *Q* = 50, which is 200 ms. For simplicity of notation, the subscript n that indicates clip number will be dropped unless otherwise stated, rendering the cochleagram tensor as $z_{fq}[t]$ and the PSTH as $y[t]$.

The encoding models

The spectro-temporal receptive field and the linear-nonlinear model

The linear-nonlinear (LN) model consists of a linear stage, the spectrotemporal receptive field (STRF), and a non-linear stage. The linear part of the model is:

$$a[t] = \sum_{f,q} w_{fq} z_{fq}[t] + b \; \ldots \; (2)$$

where w is a vector of the input weights and *b* is the background activity of the neuron. Both *w* and *b* are free parameters of the model, which were estimated by linear regression of the neuron's firing rate $y[t]$ on the cochleagram tensor $z_{fq}[t]$ using glmnet (28), where $a[t]$ is the linear estimate of $y[t]$. To overcome overfitting, the parameters were regularized using L_1-norm (LASSO) regularization of the

weights. A regularization hyperparameter λ was used to control the strength of the regularization, which was chosen by a crossvalidation procedure (see below).

The second stage of the model was a logistic activation function (sigmoid nonlinearity), which was fitted after fitting the STRF. This function is given by:

$$\hat{y}[t] = \frac{\rho_1}{1+\exp\left(\frac{-a[t]-\rho_3}{\rho_2}\right)} + \rho_4 \; \ldots \; (3)$$

The four parameters ρ_i of the function were fitted by minimizing the squared error between the nonlinear estimate of the firing rate $\hat{y}[t]$ and measured firing rate $y[t]$.

The network receptive field model

The network receptive field (NRF) models the neural response using a network, and is the same model as reported in (1) and a slightly modified form of (2). This consists of an artificial neural network with a single hidden layer of 20 hidden units (HU) that converge onto an output unit (OU). Each hidden unit of the model is similar to a single LN model, which all then feed into another LN model that predicts the neural response. The activation of the *j*-th HU, $a_j[t]$ is given by,

$$a_j[t] = \sum_{f,q} w_{jfq} z_{fq}[t] + b_j \; \ldots \; (4)$$

The output of the *j*-th HU, $v_j[t]$ is,

$$v_j[t] = \mathrm{g}\left(a_j[t]\right) \; \ldots \; (5)$$

where $g(a_j[t])$ is a sigmoid non-linear activation function, $1/(1+\exp(-a_j[t]))$. The HUs feed these outputs to the OU. The activation function of the OU, $a_o(t)$ is,

$$a_o[t] = \sum_j w_j v_j[t] + b_o \ \ldots \ (6)$$

The output $v_o[t]$ of the OU is,

$$v_o[t] = g(a_o[t]) \ \ldots \ (7)$$

which is the model's estimate of the neuron's response at time *t*.

The free parameters (the weights w_{jfq} and w_j, and the biases b_j and b_o) were optimized by minimizing the squared error between predicted response, $v_o[t]$, and actual response, *y*[*t*], subject to L_1-regularization of the weights. Thus, the objective function is given by,

$$E = \frac{1}{2NT}\sum_{n,t}\left(v_{o,n}[t] - y_n[t]\right)^2 + \lambda\left(\sum_{j,f,q}\left|w_{jfq}\right| + \sum_j\left|w_j\right|\right) \ \ldots \ (8)$$

Here, *n* is included to indicate sound clip number, but was left out of other equations for simplicity. *N* is the number of clips used in training.

The parameters of the NRF model are fitted by minimizing the objective function with respect to the free parameters using the sum-of-function optimizer algorithm (29). In using this algorithm, we take one clip to be one minibatch. The optimization algorithm requires calculation of the error gradients in respect to

each of the parameters. For the NRF model, error gradients are calculated using standard chain rule (backpropagation).

Before training, the weights were initialized by modified Glorot initialization from a uniform distribution ranging from - $\frac{1}{\sqrt{FQ+L}}$ to + $\frac{1}{\sqrt{FQ+L}}$, where FQ is the number of input weights to a HU and L = 1 is the number of output weights from a HU. The biases were initialized similarly (30).

Cross-validation and testing of the encoding models

Each sound dataset was divided into a cross-validation set and a test set. The cross-validation set was used for training the weight matrices of the encoding models and setting their regularization hyperparameter strength by cross-validation. Also, for all cochlear models, biologically-complex and simple, cross-validation methods (using NS1 mostly) were used to explore some of their settings to avoid using any settings that gave poor predictions. The hyperparameters and settings were selected only using the cross-validation set, and then the separate test set was used to assess the prediction capacity of the models.

More specifically, for the natural sound dataset 1, which contained 20 sound clips, 4 were chosen as a test set that was not used during training and cross-validation. The cross-validation set (the remaining 16 clips) was used to fit the models using *k*-fold cross-validation, where *k* = 8. The cross-validation set was randomly divided into a training set of 14 clips and a validation set of 2 clips. The encoding models were trained on the training set for 18 different values of the hyperparameter λ. A log spaced range of lambda values was used, but with a somewhat lower density at the extremes. For the LN model, the exact values of λ used were: 1.00 x 10^{-1}, 2.00 x 10^{-2}, 1.17 x 10^{-2}, 6.84 x 10^{-3}, 4.00 x 10^{-3}, 2.34 x 10^{-3}, 1.37 x 10^{-3}, 8.00 x 10^{-4}, 4.68 x 10^{-4}, 2.74 x 10^{-4}, 1.60 x 10^{-4}, 9.36 x 10^{-5}, 5.41 x 10^{-5}, 3.20 x 10^{-5}, 6.40 x 10^{-6}, 1.28 x 10^{-6}, 2.56 x 10^{-7}, and 5.12 x 10^{-8}. For the NRF model, the exact values of λ used were: 1.00 x 10^{-3}, 2.00 x 10^{-4}, 1.17 x 10^{-4}, 6.84 x 10^{-5}, 4.00 x 10^{-5}, 2.34 x 10^{-5}, 1.37 x 10^{-5}, 8.00 x 10^{-6}, 4.68 x 10^{-6}, 2.74 x 10^{-6}, 1.60 x 10^{-6}, 9.36 x 10^{-7}, 5.41 x 10^{-7}, 3.20 x 10^{-7}, 6.40 x 10^{-8}, 1.28 x 10^{-8}, 2.56 x 10^{-9}, and 5.12 x 10^{-10}. For each LN/NRF model fitted with different λ, neural responses were then predicted for the

validation set, and the correlation coefficient between the actual neural responses and the prediction was measured. This process was repeated 8 times for different non-overlapping validation sets, so the whole cross-validation set provided validation. The model was then retrained with the whole cross-validation set using the λ value that provided the highest mean correlation coefficient over all 8 folds. Next, the retrained model was used to predict the neural responses to the test set. All the correlation coefficients and normalized correlation coefficients shown are for this held-out test set, and all the LN model parameters shown are for the retrained model.

For the other datasets, clip numbers in the training, validation and test sets followed similar proportions. For natural sound dataset 2, which contained 18 natural sound clips, 4 were chosen as the test set and 14 as the cross-validation set. These 14 clips were then further divided into a training set (12 clips) and a validation set (2 clips) – this was repeated 7 times to obtain 7-fold cross-validation with 7 non-overlapping validation sets that spanned the cross-validation set. For the DRC dataset, among the 12 sound clips, 3 were chosen as the test set and 9 as the cross-validation set. These 9 clips were then further divided into a training set (7 clips) and a validation set (2 clips) – this was repeated 5 times to obtain 5-fold cross-validation with 5 mostly non-overlapping validation sets that spanned the cross-validation set. The range of values explored of hyperparameter λ remained unchanged among datasets.

Supplementary Figures and Tables

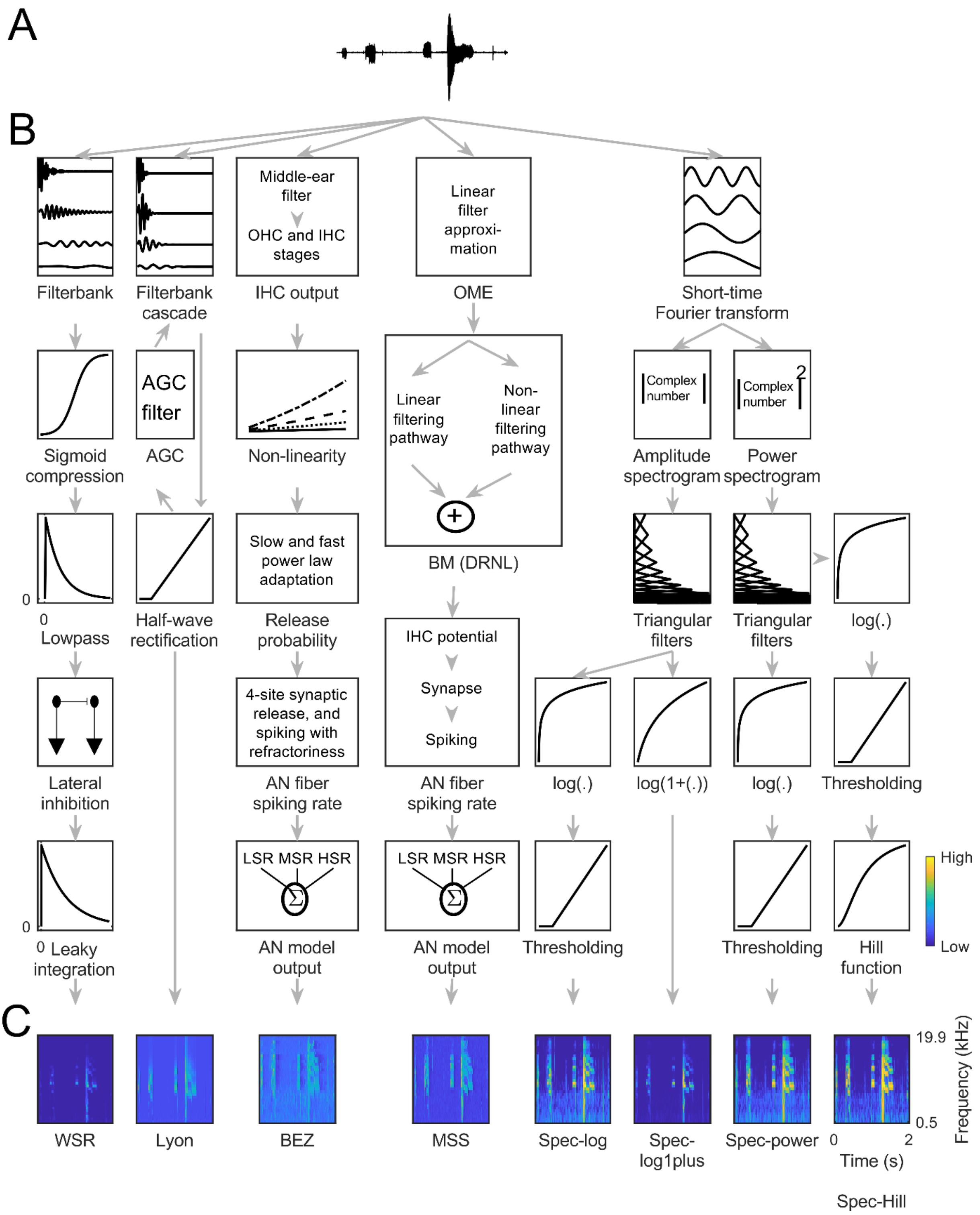

Figure S1: Schematics of the cochlear models. A. A sound waveform, the input to a cochlear model. B. The stages of transformation of sound through each of the cochlear models (from left to right): Wang Shamma Ru (WSR) model (10–13), Lyon model (14, 15), Bruce Erfani Zilani (BEZ) model (16–18), Meddis Sumner Steadman (MSS) model (19–21, 23, 24), spec-log model, spec-log1plus model, spec-

power model and spec-Hill model (see Supplementary Methods). OME, outer and middle ear; OHC, outer hair cell; IHC, inner hair cell; BM, basilar membrane; DRNL, dual resonance non-linear filter; lin, linear; nonlin, nonlinear; AN, auditory nerve; LSR, low spontaneous rate; MSR, medium spontaneous rate; HSR, high spontaneous rate. C. The output of the cochlear models, the cochleagram. The example shown here is a 3 s excerpt of a sound of a wolf howling by a waterfall.

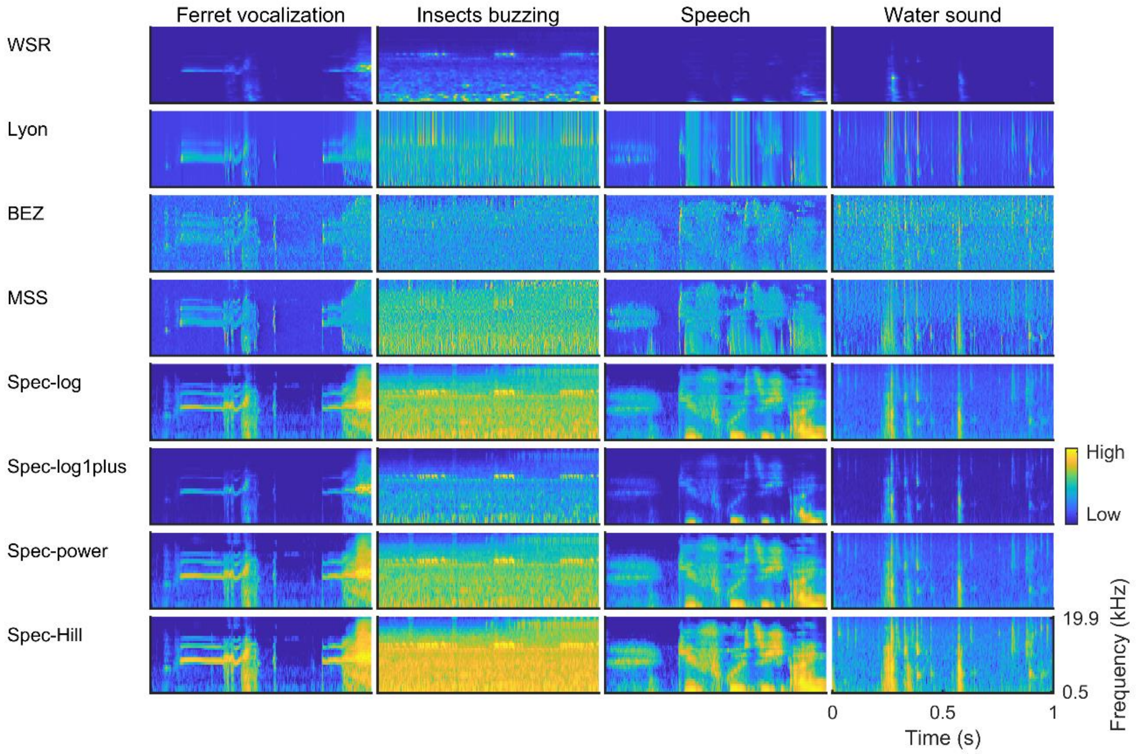

Figure S2: Examples of cochleagrams of natural sound stimuli. Cochleagrams of 1 s excerpts of various natural sound clips are shown here. All cochleagrams contain 32 frequency channels. Only very high energy regions are visible in WSR cochleagrams since its compressive function does not convert sound energy to a range that is distributed evenly over the colormap.

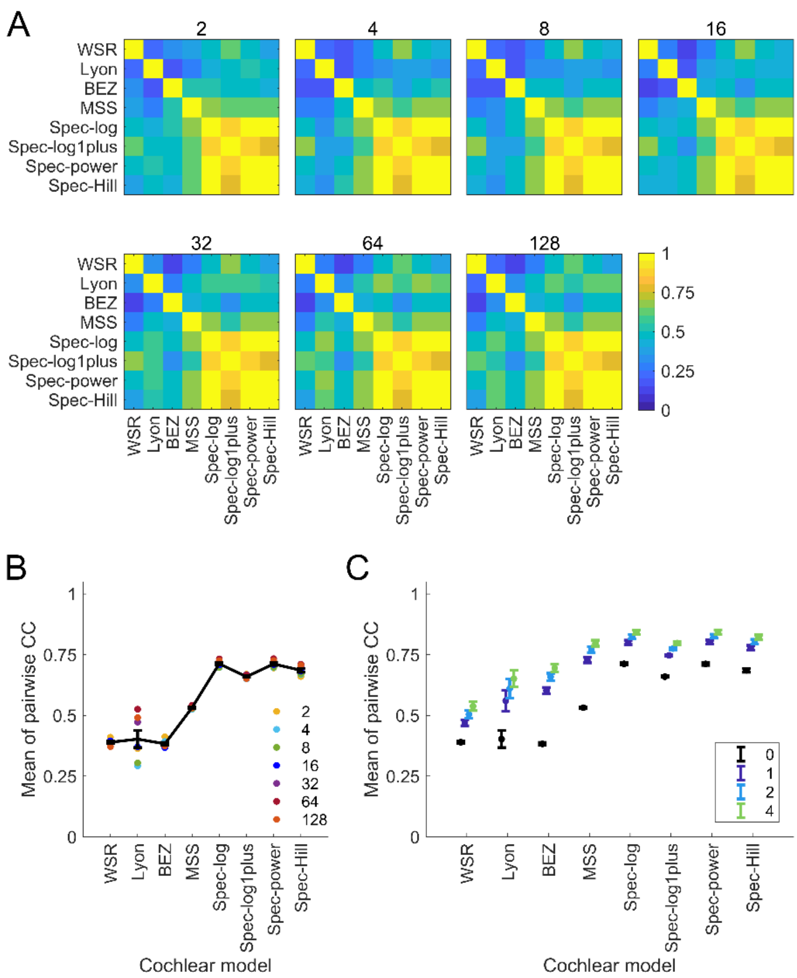

Figure S3: Comparison of cochleagrams produced by different models. A. Correlation coefficient (CC) between cochleagrams produced by all possible pairs of models. The number on top of each plot shows the number of frequency channels in the cochleagram. B. Mean of the pair-wise CC of a model with the rest of the models. Colored dots show the mean for a model with a specified number of frequency channels and the black line with error bars show the mean over the means for different frequency channel numbers. C. Mean of the means of the pair-wise CCs after introducing Gaussian blurring to each cochleagram to account for spectral and temporal shift.

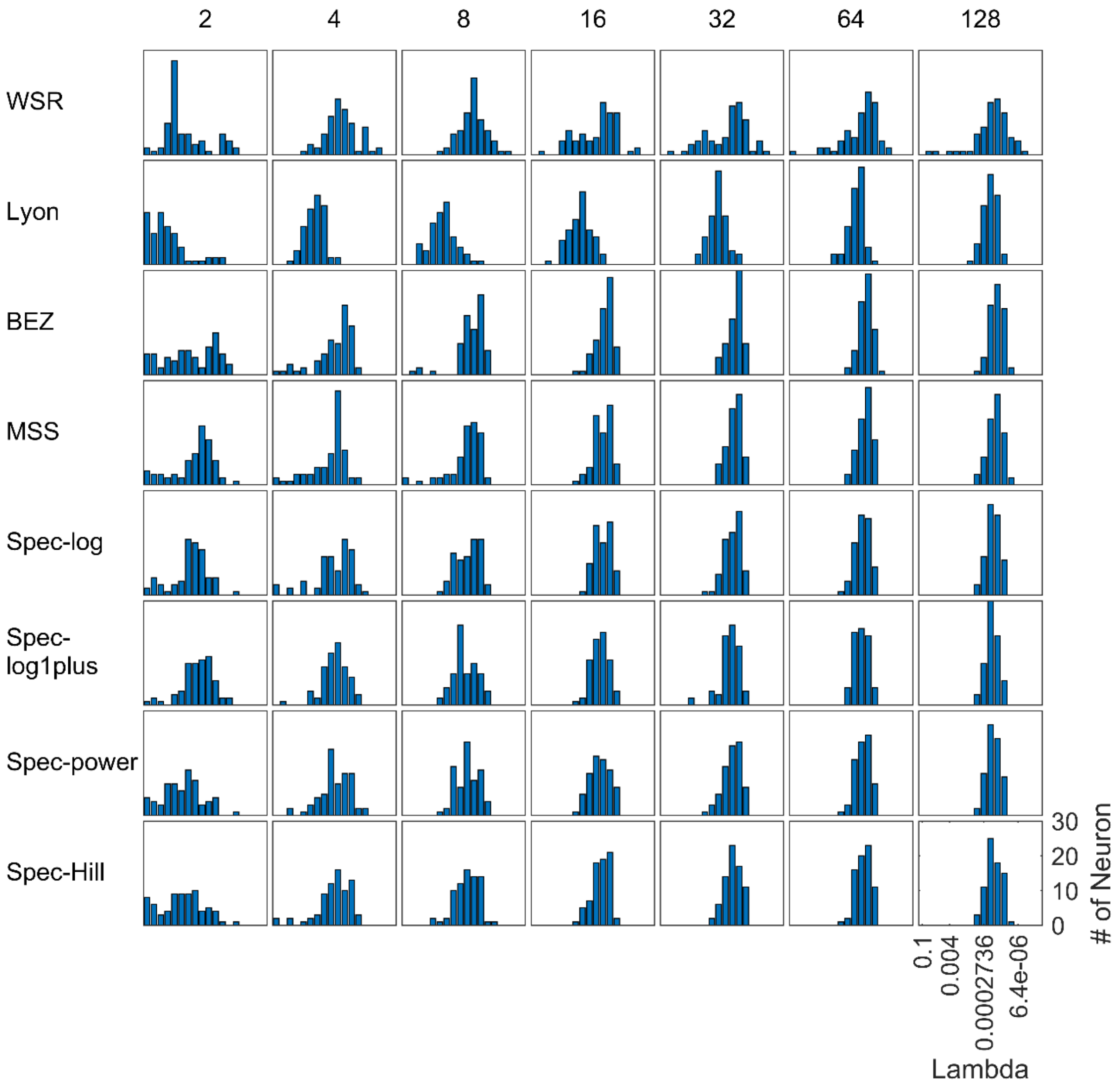

Figure S4: Distribution of the values of hyperparameter lambda in the LN model across the cortical neurons. Each row shows the cochlear model that was used to generate the input for the LN model and each column shows the number of frequency channels in the cochleagram input.

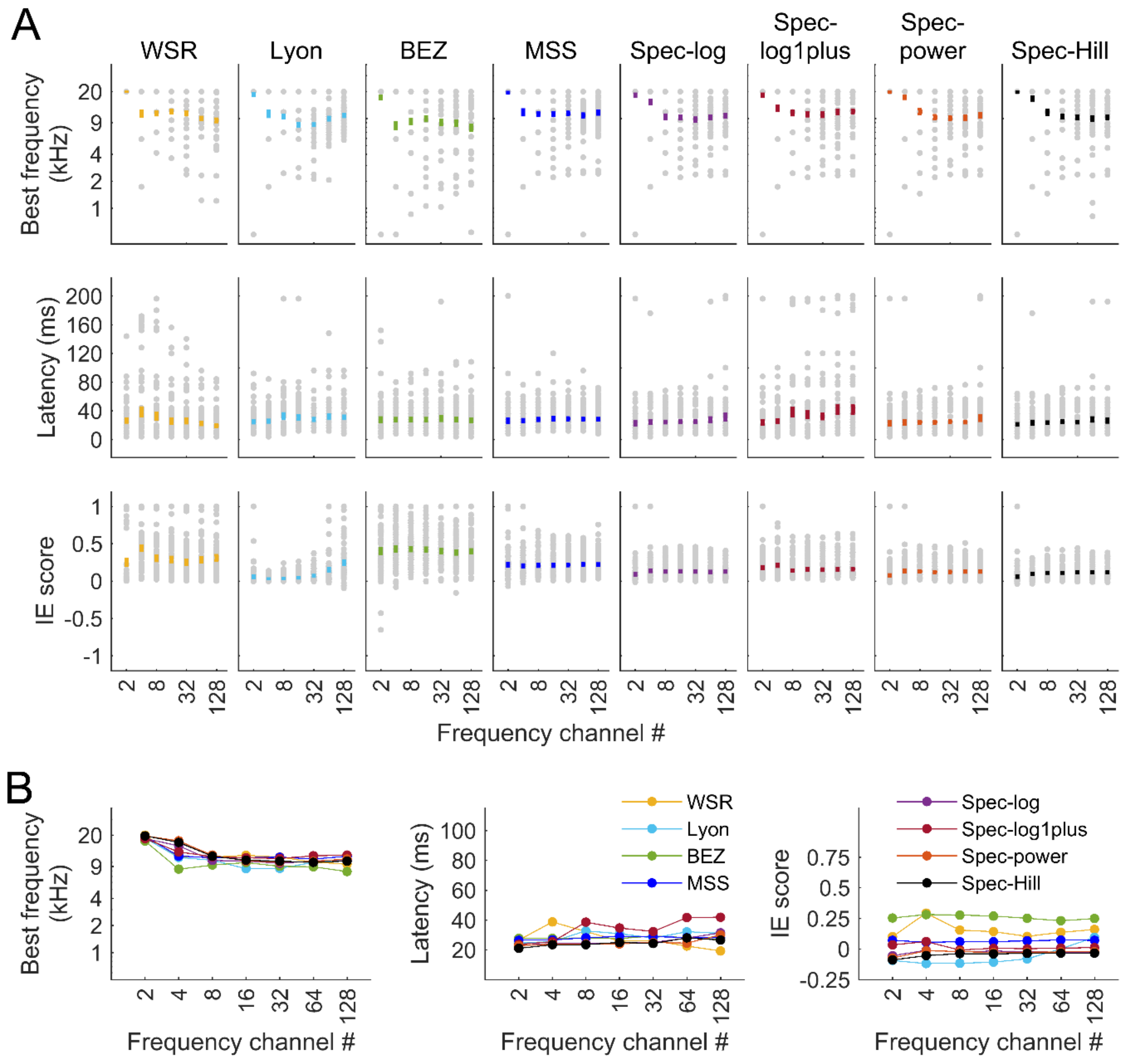

Figure S5: Cortical response properties estimated by using different cochleagram inputs to the LN model. A. Best frequency, latency and IE score are shown in the top, middle and bottom rows, respectively, with each plot showing the estimated values using the model specified at the top and the number of frequency channels specified on the x-axis. Each gray dot is one neuron and the color bar indicates the mean and the squared error of the mean. B. Mean best frequency, latency and IE score over all neurons for each model and number of frequency channels.

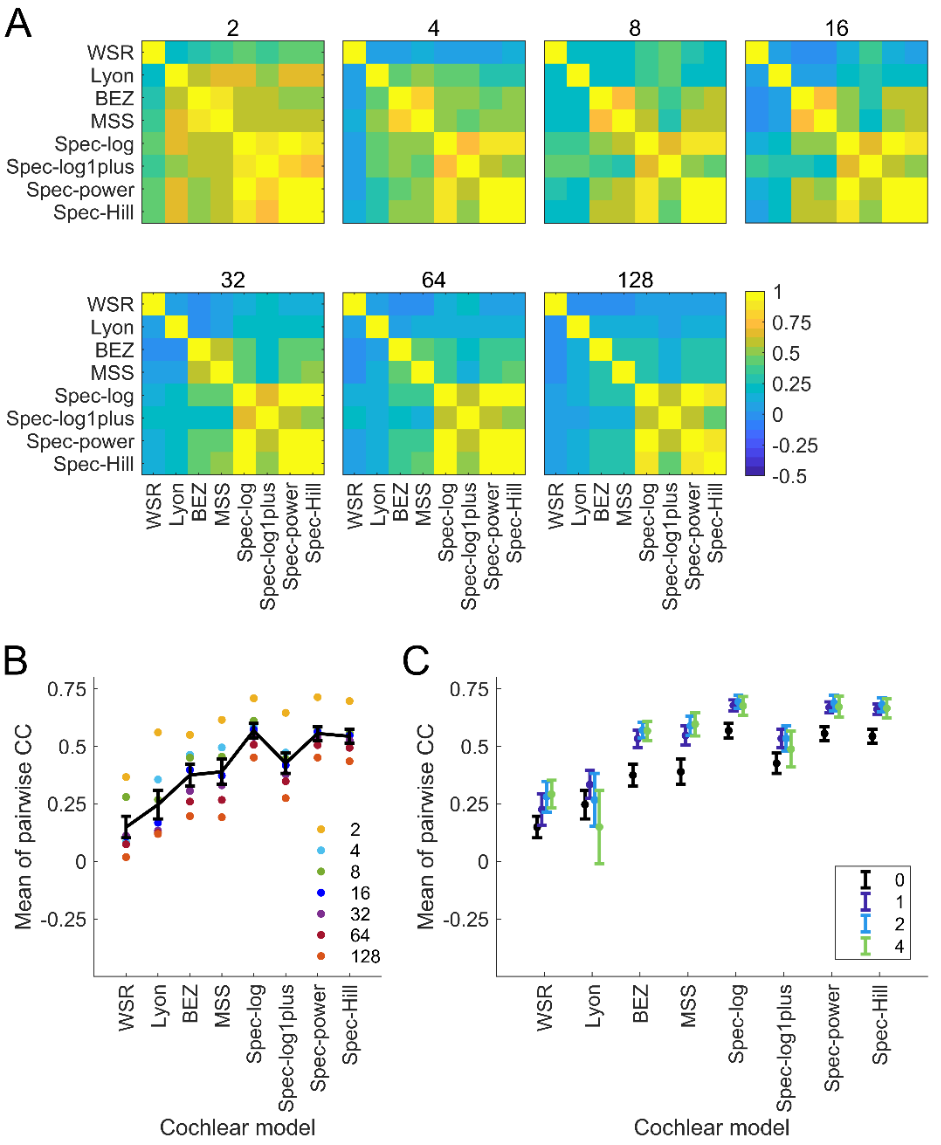

Figure S6: Comparison of STRFs estimated using with different cochleagram inputs. A. Correlation coefficient (CC) between STRFs produced by all possible pairs of cochlear models. The number on top of each plot shows the number of frequency channels in the cochleagram. B. For each model, the mean CC of its STRFs with the STRFs of the other models with the same number of frequency channels. Colored dots show the mean for a model with a specified number of frequency channels and the black line with error bars shows the mean over the means for different numbers of frequency channels. C. Mean of the means of the pair-wise CCs after introducing Gaussian blurring to each STRF to account for spectral and temporal shift.

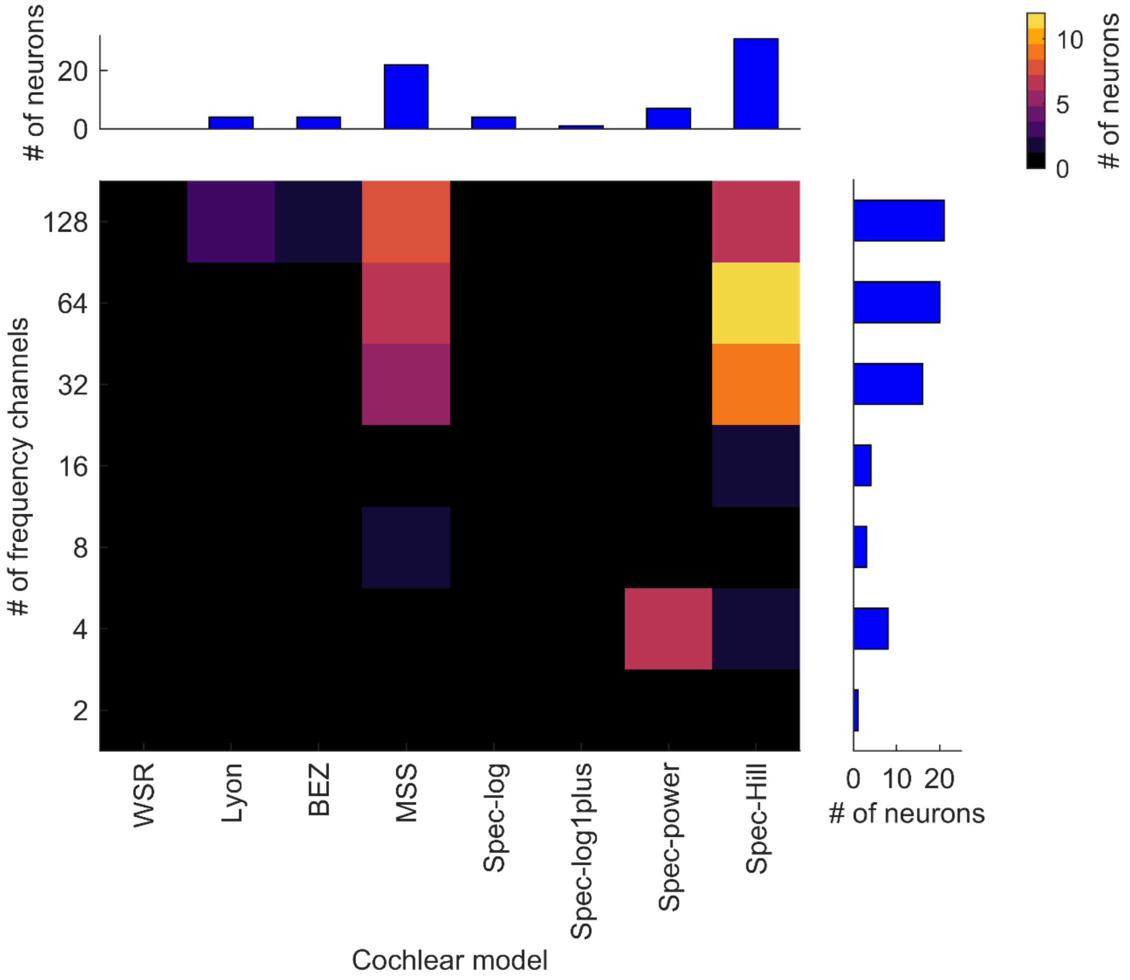

Figure S7: Best performing cochlear models for 73 primary auditory cortical neurons. The heatmap shows the number of neurons for which each model with a specific number of frequency channels is the best performing model and the bars show the data collapsed along each axis.

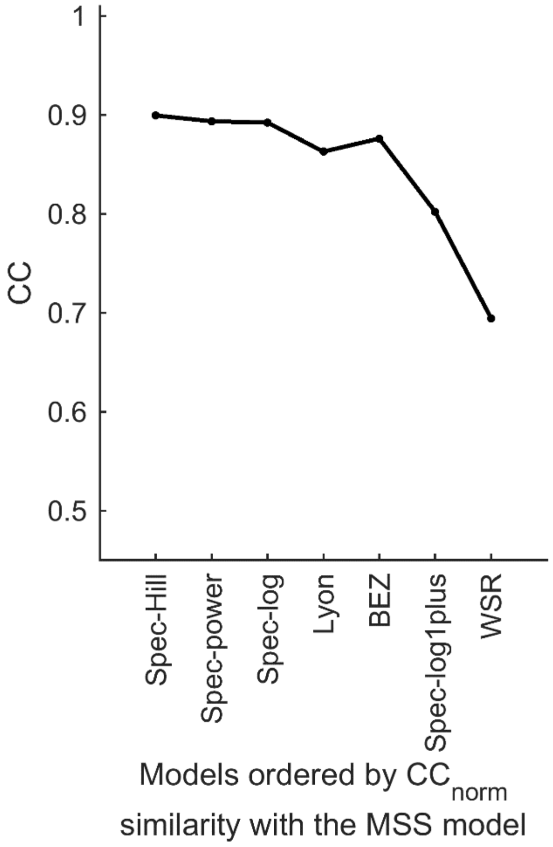

Figure S8: Similarity of the predicted response of the MSS model to the predicted response of other models. Each dot represents the correlation co-efficient between the response of the MSS model and the specified model in the x-axis. Models on the x-axis are ordered by similarity of their CC_{norm} performance to show the relationship between prediction performance and response similarity.

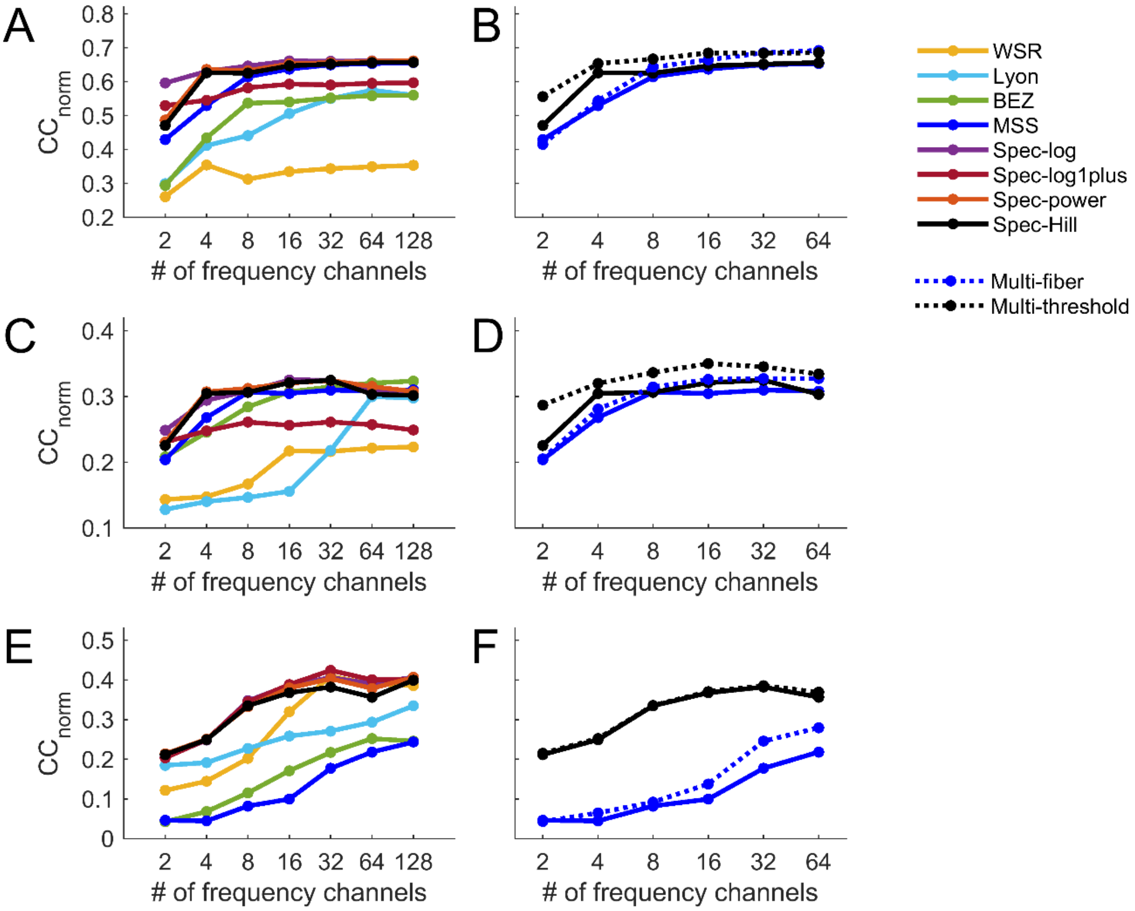

Figure S9: Average CC_{norm} for output of linear stage of the LN encoding model for all three datasets. A and B. CC_{norm} on the natural sound dataset 1. C and D. CC_{norm} on the natural sound dataset 2. E and F. CC_{norm} on the DRC dataset.

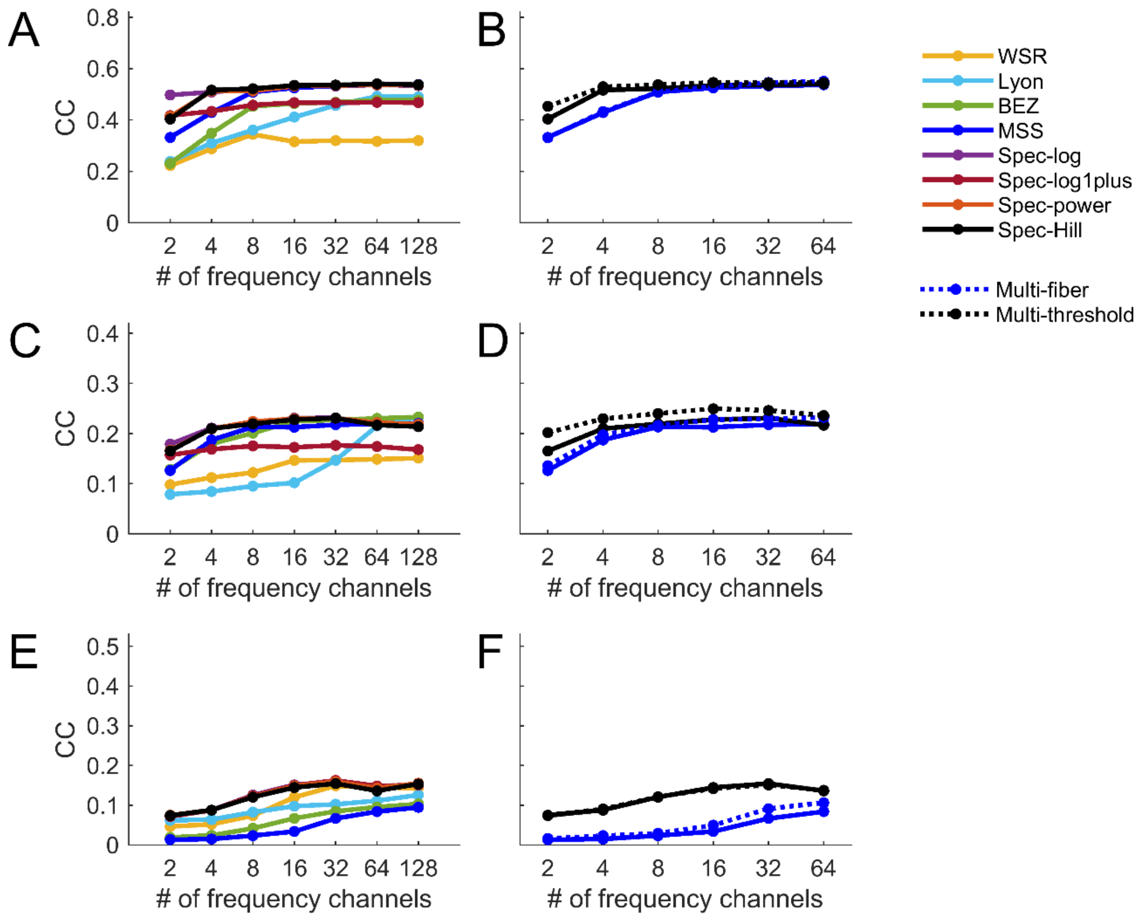

Figure S10: Prediction accuracy of models when correlation coefficient (CC) is used as performance measure. A and B. CC on the natural sound dataset 1. C and D. CC on the natural sound dataset 2. E and F. CC on the DRC dataset.

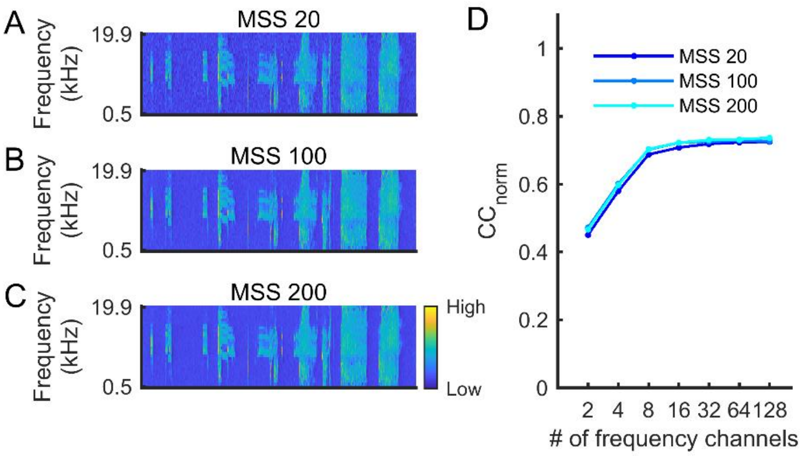

Figure S11: Effect of noise on the prediction performance of the MSS model. A. Cochleagram of a speech sound produced by an MSS model averaged over 20 repeats (MSS 20). B. Cochleagram of the same sound produced by an MSS model averaged over 100 repeats (MSS 100) and C. 200 repeats (MSS 200). D. Comparison of the prediction performance of the MSS 20, MSS 100 and MSS 200 models.

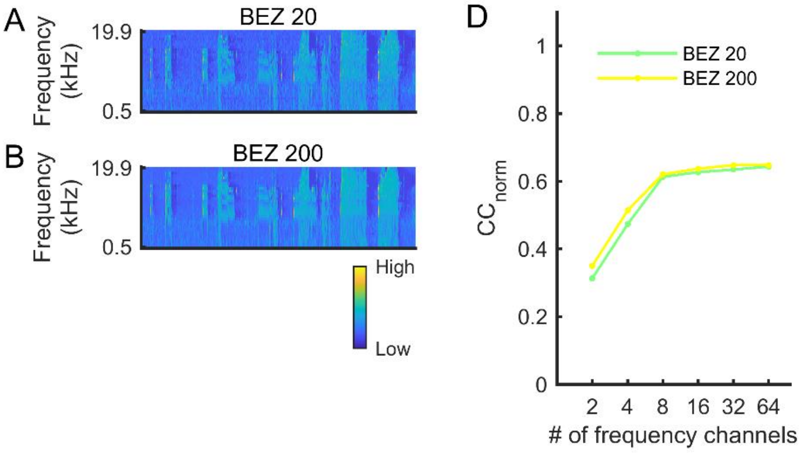

Figure S12: Effect of noise on the prediction performance of the BEZ model. A. Cochleagram of a speech sound produced by a BEZ model averaged over 20 repeats (BEZ 20). B. Cochleagram of the same sound produced by a BEZ model averaged over 200 repeats (BEZ 200) and C. Comparison of the prediction performance of the MSS 20 and the BEZ 200 model. The BEZ model takes much longer to run than the other model. For the sake of running time, we ran the BEZ 200 model only up to 64 frequency channels.

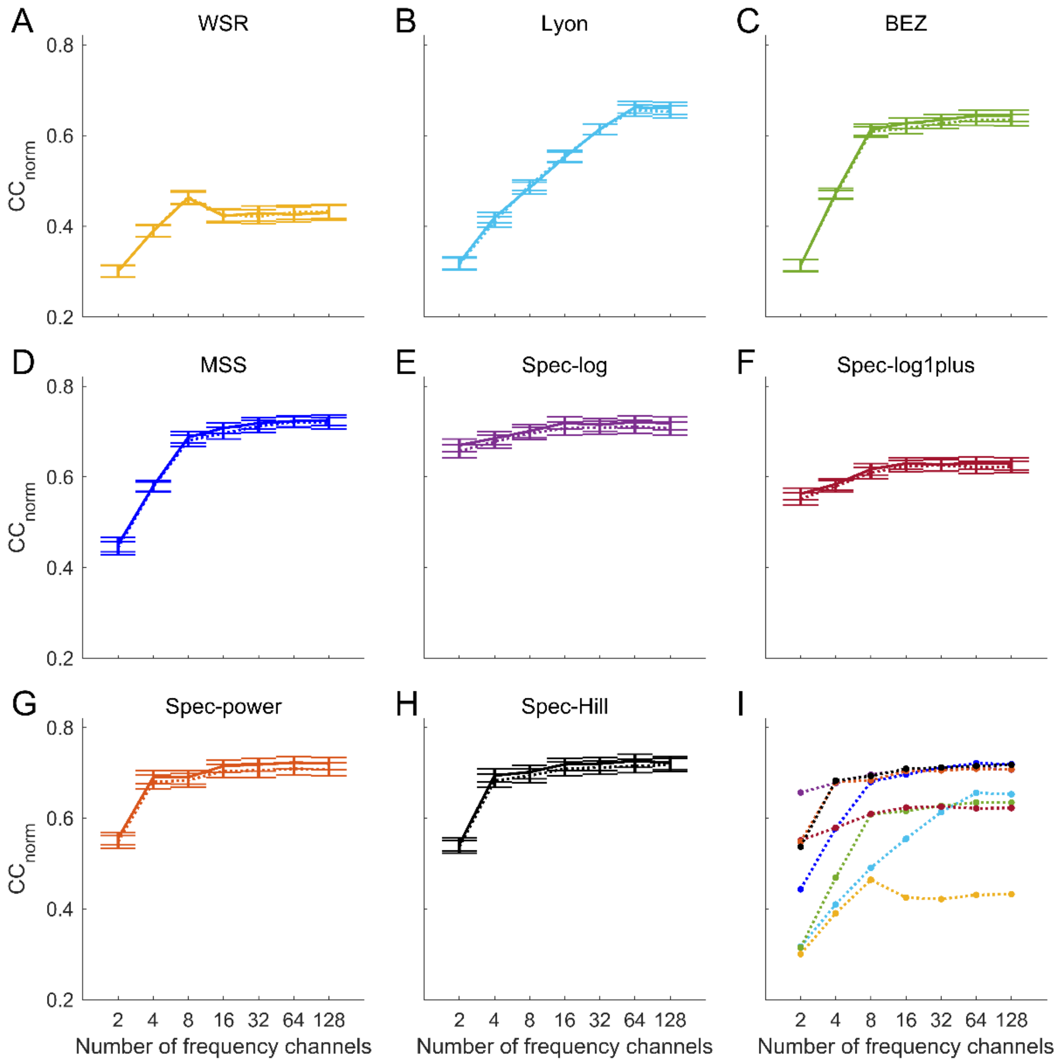

Figure S13: Average CC_{norm} of different cochlear models when onset response is included for prediction. A-H. Solid lines are without onset response (without 0-800 ms), as in Figure 3. Dotted lines are with the onset response included. Error bars on solid and dotted lines are the standard error. I. The CC_{norm} of all the different cochlear models with onset response included.

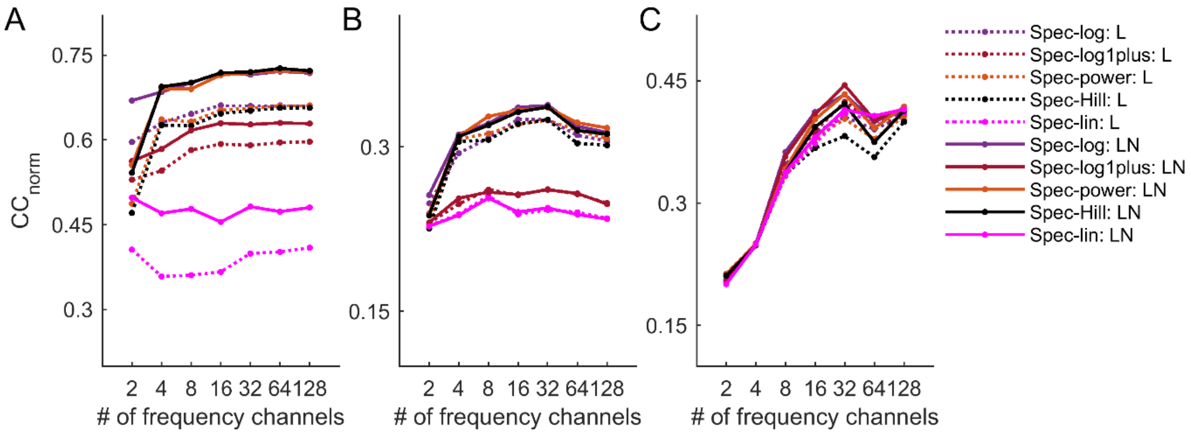

Figure S14: Interaction between compressive cochlear non-linearities and LN-model output non-linearities. Average CC_{norm} of spectrogram-based models (spec-lin is the spectrogram-based model without any compressive cochlear non-linearity) for an LN encoding model with (LN) and without (L) the output non-linearity. A. CC_{norm} on the natural sound dataset 1. B. CC_{norm} on the natural sound dataset 2. C. CC_{norm} on the DRC dataset.

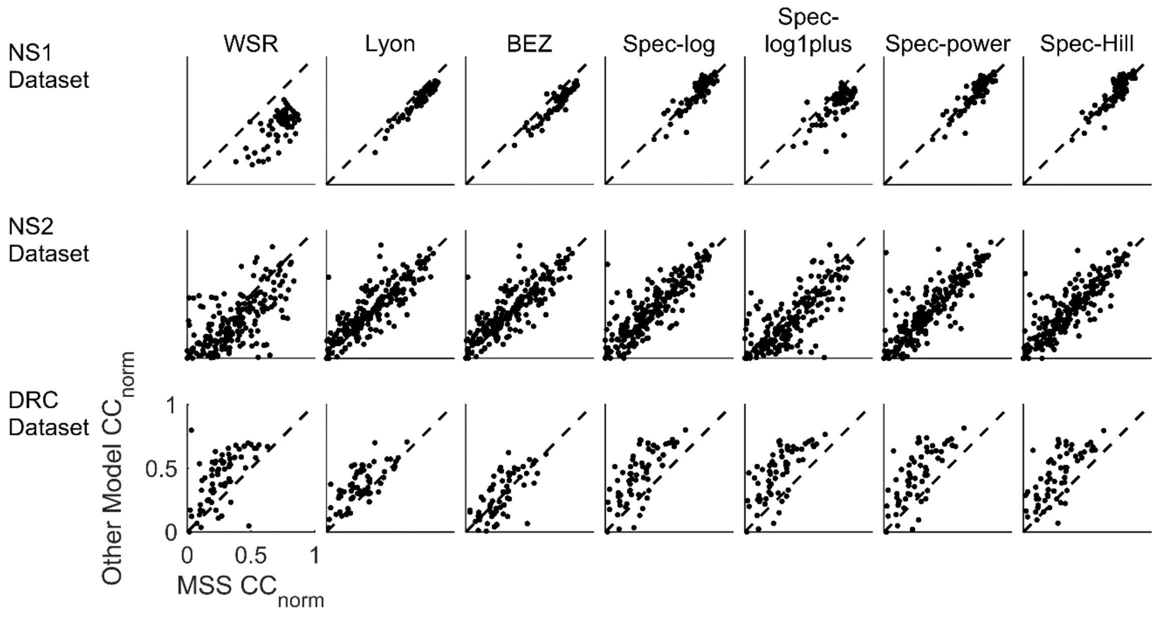

Figure S15: Neuron by neuron comparison of model performance. Each model with its optimum number of frequency channels was chosen for this analysis. Each panel shows the scatterplot of the CC_{norm} of each neuron for a model (model name on top) versus the MSS model. Rows are different datasets. Each dot is a neuron.

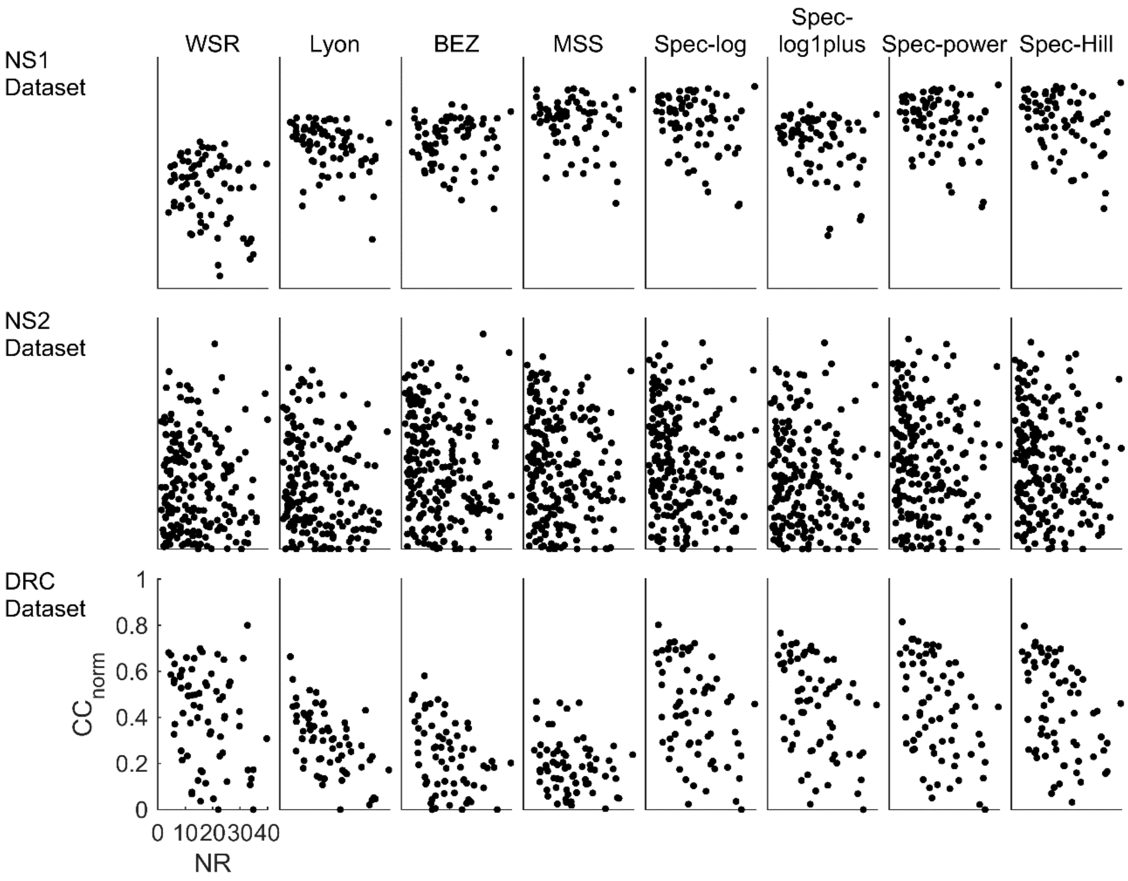

Figure S16: CC_{norm} as a function of noise ratio, for the three datasets and all the models. Each panel shows the scatterplot of the CC_{norm} of each neuron for a model (model name on top) versus the noise ratio (NR) of each neuron. Each model with 32 frequency channels was chosen for this analysis. Rows are different datasets. Each dot is a neuron.

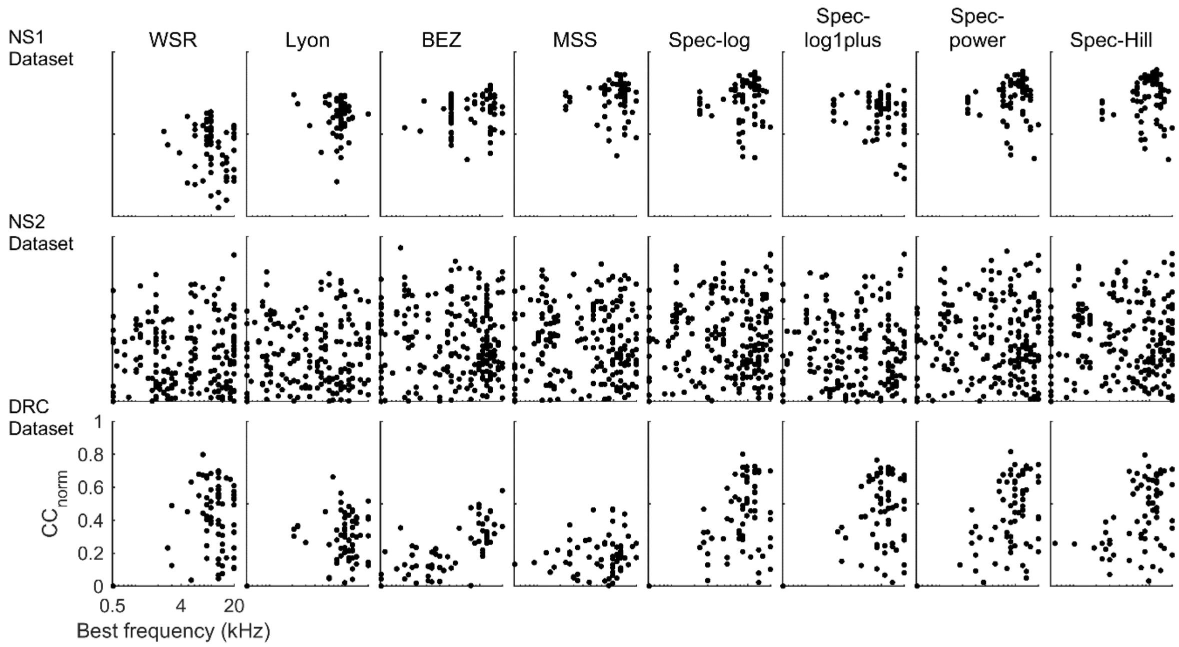

Figure S17: CC_{norm} as a function of best frequency of the STRF, for the three datasets, and all the models. Each panel shows the scatterplot of the CC_{norm} of each neuron for a model (model name on top) versus the best frequency of each neuron estimated from the STRF. Best frequency was measured as the frequency field that produced the maximum absolute value in the STRF of a neuron. Each model with 32 frequency channels was chosen for this analysis. Rows are different datasets. Each dot is a neuron.

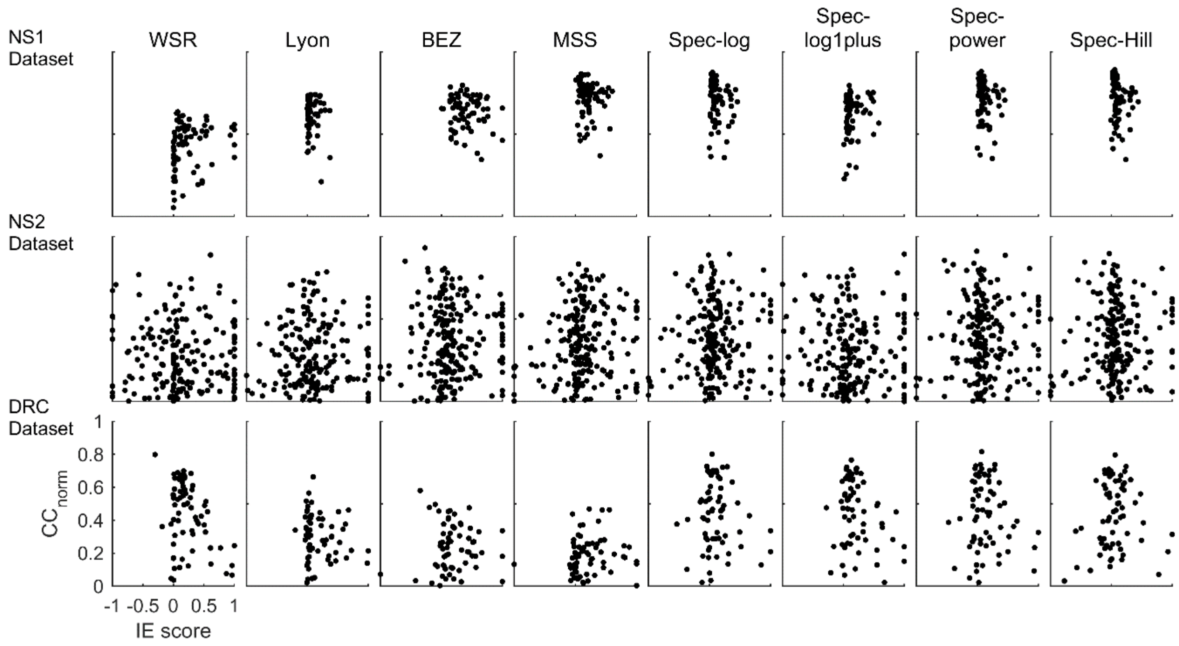

Figure S18: CC_{norm} as a function of IE score of the STRF for the three datasets, and all the models. IE scores measures the difference in strength of excitatory (positive) weights and inhibitory (negative) weights in an STRF (1, 2). A value of 1 indicates all excitatory weights, a value of -1 indicates all inhibitory weights. Each panel shows the scatterplot of the CC_{norm} of each neuron for a model (model name on top) versus the BF of each neuron estimated from the STRF. Each model with 32 frequency channels was chosen for this analysis. Rows are different datasets. Each dot is a neuron.

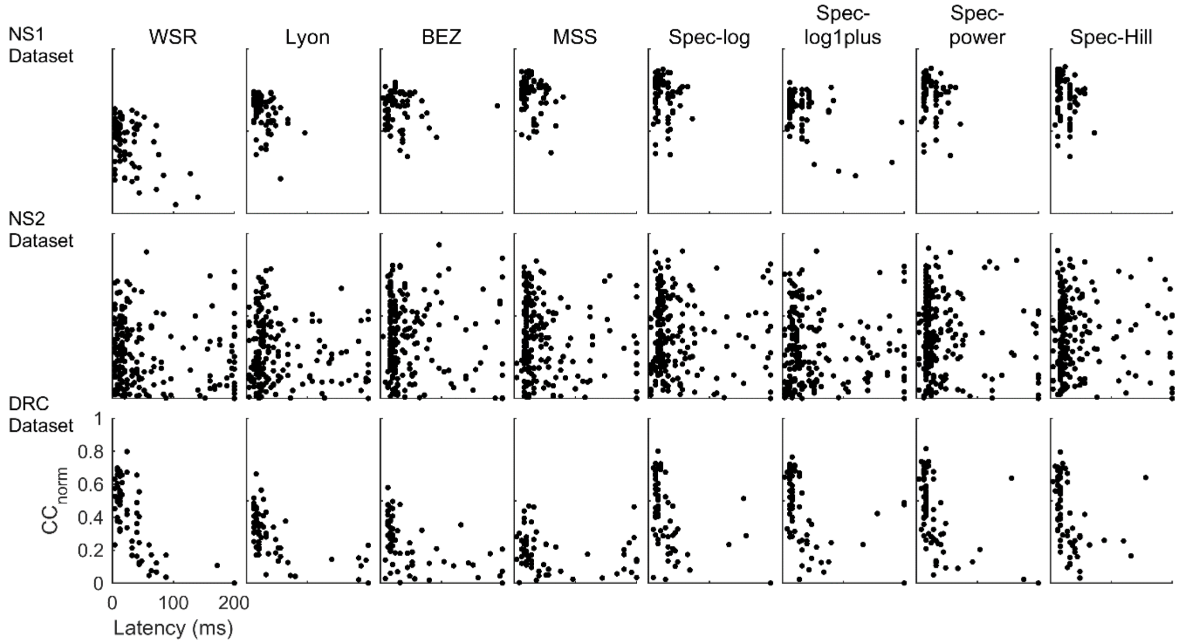

Figure S19: CC_{norm} as a function of noise ratio for the latency of the STRF, and all the models. Each panel shows the scatterplot of the CC_{norm} of each neuron for a model (model name on top) versus the latency of each neuron estimated from the STRF. Latency was measured as the time delay that had maximum absolute value in the STRF of a neuron. Each model with 32 frequency channels was chosen for this analysis. Rows are different datasets. Each dot is a neuron.

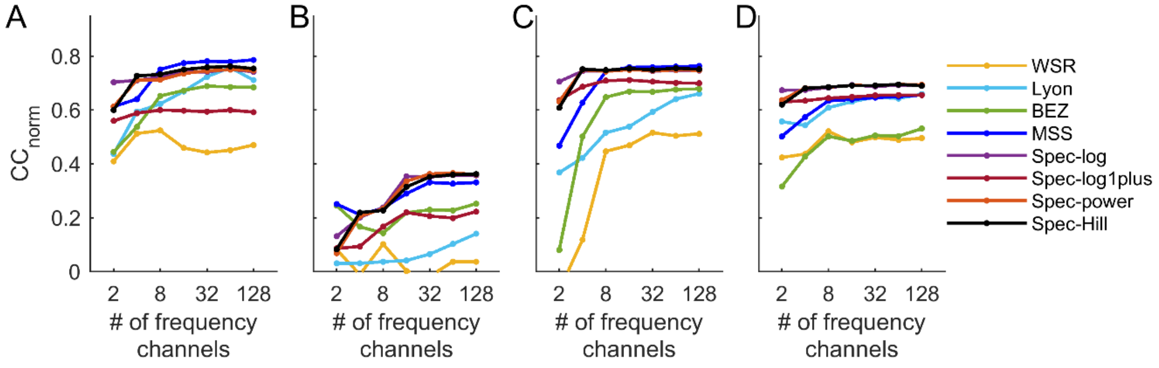

Figure S20: CC_{norm} as a function of number of frequency channels, for each cochlear model, for each stimulus type in the test set. A. Ferret vocalizations. B. Other animal sounds. C. Speech. D. Environmental sounds.

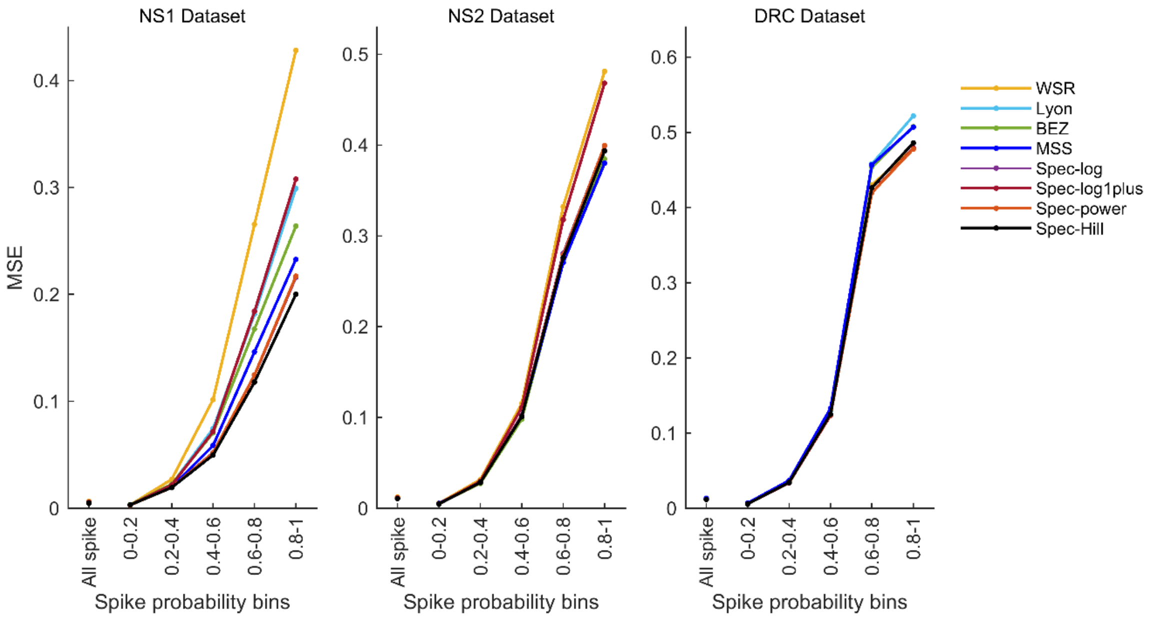

Figure S21: Mean squared error (MSE) of model predictions as a function of recorded spike probability. Each panel is a different dataset, each color is a different cochlear model. For each dataset and model, the MSE between the model prediction and the real PSTH was measured, as function of the spike probability in the PSTH. “All spike” is the MSE over all spike probabilities.

Table S1: Mean ± SE CC_{norm} between cortical responses and predicted responses for the LN model.

	Number of frequency channels						
**Models**	**2**	**4**	**8**	**16**	**32**	**64**	**128**
**Mean CC_{norm} of the models for the natural sound dataset 1**							
**WSR**	0.301 ± 0.013	0.390 ± 0.013	0.462 ± 0.014	0.423 ± 0.015	0.429 ± 0.017	0.426 ± 0.016	0.430 ± 0.016
**Lyon**	0.320 ± 0.013	0.419 ± 0.012	0.485 ± 0.013	0.552 ± 0.012	0.614 ± 0.012	0.662 ± 0.013	0.660 ± 0.014
**BEZ**	0.313 ± 0.013	0.473 ± 0.011	0.613 ± 0.013	0.627 ± 0.012	0.635 ± 0.012	0.643 ± 0.012	0.644 ± 0.013
**MSS**	0.451 ± 0.015	0.580 ± 0.012	0.687 ± 0.012	0.708 ± 0.012	0.719 ± 0.012	0.723 ± 0.012	**0.725 ± 0.012**
**Spec-log**	0.670 ± 0.014	0.685 ± 0.014	0.701 ± 0.014	0.719 ± 0.014	0.715 ± 0.014	0.721 ± 0.014	0.718 ± 0.014
**Spec-log1plus**	0.562 ± 0.013	0.584 ± 0.013	0.617 ± 0.012	0.630 ± 0.013	0.627 ± 0.014	0.630 ± 0.013	0.629 ± 0.013
**Spec-power**	0.556 ± 0.013	0.690 ± 0.014	0.690 ± 0.015	0.715 ± 0.014	0.718 ± 0.014	0.722 ± 0.014	0.720 ± 0.014
**Spec-Hill**	0.542 ± 0.014	0.694 ± 0.015	0.701 ± 0.015	0.719 ± 0.014	0.720 ± 0.014	**0.726 ± 0.014**	0.722 ± 0.014
**Multi-fiber**	0.450 ± 0.012	0.584 ± 0.011	0.688 ± 0.011	0.716 ± 0.011	0.736 ± 0.012	**0.742 ± 0.012**	
**Multi-threshold**	0.610 ± 0.012	0.713 ± 0.012	0.724 ± 0.013	0.736 ± 0.012	0.734 ± 0.012	0.734 ± 0.012	
**Mean CC_{norm} of the models for the natural sound dataset 2**							
**WSR**	0.150 ± 0.015	0.172 ± 0.015	0.183 ± 0.016	0.219 ± 0.015	0.219 ± 0.014	0.222 ± 0.014	0.224 ± 0.015
**Lyon**	0.120 ± 0.013	0.127 ± 0.013	0.142 ± 0.013	0.152 ± 0.014	0.214 ± 0.015	0.308 ± 0.017	0.340 ± 0.015
**BEZ**	0.197 ± 0.014	0.263 ± 0.014	0.298 ± 0.015	0.328 ± 0.015	0.332 ± 0.015	0.336 ± 0.015	0.340 ± 0.015
**MSS**	0.194 ± 0.014	0.276 ± 0.014	0.314 ± 0.015	0.309 ± 0.016	0.315 ± 0.016	0.319 ± 0.015	0.319 ± 0.015
**Spec-log**	0.256 ± 0.016	0.312 ± 0.017	0.322 ± 0.016	0.336 ± 0.016	0.338 ± 0.016	0.319 ± 0.017	0.313 ± 0.016
**Spec-log1plus**	0.231 ± 0.015	0.253 ± 0.016	0.259 ± 0.016	0.257 ± 0.016	0.261 ± 0.015	0.258 ± 0.015	0.248 ± 0.015
**Spec-power**	0.239 ± 0.015	0.310 ± 0.017	0.328 ± 0.016	0.333 ± 0.016	0.336 ± 0.016	0.322 ± 0.016	0.317 ± 0.016
**Spec-Hill**	0.238 ± 0.015	0.310 ± 0.017	0.320 ± 0.016	0.332 ± 0.016	0.336 ± 0.016	0.315 ± 0.016	0.312 ± 0.016

**Multi-fiber**	0.207 ± 0.014	0.289 ± 0.014	0.318 ± 0.015	0.332 ± 0.015	0.333 ± 0.015	0.338 ± 0.015	
**Multi-threshold**	0.297 ± 0.015	0.335 ± 0.016	0.352 ± 0.016	0.364 ± 0.016	0.358 ± 0.016	0.344 ± 0.016	
**Mean CC_{norm} of the models for the DRC dataset**							
**WSR**	0.113 ± 0.018	0.138 ± 0.019	0.197 ± 0.019	0.323 ± 0.024	0.411 ± 0.025	0.400 ± 0.025	0.397 ± 0.026
**Lyon**	0.174 ± 0.014	0.185 ± 0.015	0.228 ± 0.017	0.264 ± 0.018	0.272 ± 0.020	0.302 ± 0.020	0.344 ± 0.020
**BEZ**	0.038 ± 0.012	0.057 ± 0.012	0.105 ± 0.013	0.165 ± 0.017	0.212 ± 0.019	0.250 ± 0.021	0.264 ± 0.023
**MSS**	0.037 ± 0.008	0.040 ± 0.010	0.072 ± 0.009	0.097 ± 0.012	0.182 ± 0.014	0.226 ± 0.017	0.251 ± 0.019
**Spec-log**	0.206 ± 0.017	0.250 ± 0.020	0.363 ± 0.019	0.412 ± 0.024	0.433 ± 0.025	0.400 ± 0.025	0.413 ± 0.026
**Spec-log1plus**	0.204 ± 0.017	0.248 ± 0.020	0.357 ± 0.019	0.409 ± 0.024	0.445 ± 0.025	0.402 ± 0.026	0.414 ± 0.025
**Spec-power**	0.213 ± 0.016	0.250 ± 0.019	0.342 ± 0.020	0.403 ± 0.023	0.433 ± 0.026	0.392 ± 0.024	0.418 ± 0.027
**Spec-Hill**	0.211 ± 0.016	0.249 ± 0.019	0.338 ± 0.020	0.393 ± 0.023	0.422 ± 0.025	0.375 ± 0.022	0.413 ± 0.027
**Multi-fiber**	0.041 ± 0.010	0.061 ± 0.010	0.086 ± 0.013	0.143 ± 0.013	0.251 ± 0.018	0.289 ± 0.021	
**Multi-threshold**	0.215 ± 0.015	0.255 ± 0.019	0.343 ± 0.019	0.384 ± 0.023	0.406 ± 0.025	0.373 ± 0.023	

Table S2: Mean ± SE CC_{norm} between cortical responses and predicted responses for the NRF model.

**Number of frequency channels**			
**Models**	**8**	**16**	**32**
**Mean CC_{norm} of the models for the natural sound dataset 1**			
WSR	0.469 ± 0.016	0.476 ± 0.016	0.489 ± 0.017
Lyon	0.484 ± 0.014	0.524 ± 0.014	0.635 ± 0.012
BEZ	0.634 ± 0.011	0.637 ± 0.009	0.651 ± 0.009
MSS	0.704 ± 0.010	0.718 ± 0.010	0.732 ± 0.010
Spec-log	0.723 ± 0.013	0.741 ± 0.013	0.725 ± 0.015
Spec-log1plus	0.676 ± 0.015	0.697 ± 0.014	0.699 ± 0.014
Spec-power	0.707 ± 0.013	0.740 ± 0.011	0.740 ± 0.012
Spec-Hill	0.719 ± 0.012	0.733 ± 0.012	0.739 ± 0.013
Multi-fiber	0.706 ± 0.011	0.744 ± 0.009	0.760 ± 0.010
Multi-threshold	0.761 ± 0.012	0.774 ± 0.011	**0.776 ± 0.011**
**Mean CC_{norm} of the models for the natural sound dataset 2**			
WSR	0.199 ± 0.015	0.205 ± 0.015	0.203 ± 0.016
Lyon	0.130 ± 0.012	0.141 ± 0.013	0.220 ± 0.015
BEZ	0.312 ± 0.016	0.339 ± 0.016	0.347 ± 0.016
MSS	0.314 ± 0.016	0.336 ± 0.016	0.328 ± 0.016
Spec-log	0.341 ± 0.018	0.355 ± 0.017	0.346 ± 0.017
Spec-log1plus	0.278 ± 0.016	0.263 ± 0.016	0.251 ± 0.016
Spec-power	0.353 ± 0.017	0.360 ± 0.017	0.351 ± 0.017
Spec-Hill	0.341 ± 0.017	0.333 ± 0.017	0.334 ± 0.017
Multi-fiber	0.333 ± 0.015	0.350 ± 0.015	0.351 ± 0.015
Multi-threshold	0.378 ± 0.017	0.357 ± 0.017	0.353 ± 0.017

Mean CC_{norm} of the models for the DRC dataset			
WSR	0.045 ± 0.014	0.085 ± 0.017	0.155 ± 0.022
Lyon	0.125 ± 0.016	0.123 ± 0.018	0.134 ± 0.019
BEZ	0.069 ± 0.012	0.146 ± 0.013	0.153 ± 0.016
MSS	0.058 ± 0.011	0.085 ± 0.014	0.160 ± 0.014
Spec-log	0.289 ± 0.017	0.348 ± 0.021	0.351 ± 0.022
Spec-log1plus	0.255 ± 0.024	0.297 ± 0.027	0.329 ± 0.026
Spec-power	0.232 ± 0.016	0.328 ± 0.020	0.352 ± 0.022
Spec-Hill	0.219 ± 0.014	0.313 ± 0.019	0.345 ± 0.023
Multi-fiber	0.094 ± 0.012	0.127 ± 0.017	0.237 ± 0.019
Multi-threshold	0.282 ± 0.022	0.343 ± 0.026	0.376 ± 0.027

SI References

1. M. Rahman, B. D. B. Willmore, A. J. King, N. S. Harper, A dynamic network model of temporal receptive fields in primary auditory cortex. *PLOS Comput. Biol.* **15**, e1006618 (2019).

2. N. S. Harper, *et al.*, Network receptive field modeling reveals extensive integration and multi-feature selectivity in auditory cortical neurons. *PLoS Comput. Biol.* **12**, e1005113 (2016).

3. O. Schoppe, N. S. Harper, B. D. B. Willmore, A. J. King, J. W. H. Schnupp, Measuring the performance of neural models. *Front. Comput. Neurosci.* **10**, 1–11 (2016).

4. B. D. B. Willmore, O. Schoppe, A. J. King, J. W. H. Schnupp, N. S. Harper, Incorporating midbrain adaptation to mean sound level improves models of auditory cortical processing. *J. Neurosci.* **36**, 280–289 (2016).

5. J. F. Linden, R. C. Liu, M. Sahani, C. E. Schreiner, M. M. Merzenich, Spectrotemporal structure of receptive fields in areas AI and AAF of mouse auditory cortex. *J. Neurophysiol.* **90**, 2660–2675 (2003).

6. N. C. Rabinowitz, B. D. B. Willmore, J. W. H. Schnupp, A. J. King, Contrast gain control in auditory cortex. *Neuron* **70**, 1178–1191 (2011).

7. M. L. Espejo, Z. P. Schwartz, S. V. David, Spectral tuning of adaptation supports coding of sensory context in auditory cortex. *PLoS Comput. Biol.* **15**, e1007430 (2019).

8. R. C. deCharms, D. T. Blake, M. M. Merzenich, Optimizing sound features for cortical neurons. *Science (80-.).* **280**, 1439–1444 (1998).

9. J. W. H. Schnupp, T. D. Mrsic-Flogel, A. J. King, Linear processing of spatial cues in primary auditory cortex. *Nature* **414**, 200–204 (2001).

10. K. Wang, S. Shamma, Self-normalization and noise-robustness in early auditory representations. *IEEE Trans. Speech Audio Process.* **2**, 421–435 (1994).

11. K. Wang, S. A. Shamma, Auditory analysis of spectro-temporal information in acoustic signals. *IEEE Eng. Med. Biol. Mag.* **14**, 186–194 (1995).

12. T. Chi, P. Ru, S. A. Shamma, Multiresolution spectrotemporal analysis of complex sounds. *J. Acoust. Soc. Am.* **118**, 887–906 (2005).

13. P. Ru, “Multiscal multirate spectro-temporal auditory model,” University of Maryland College Park, Maryland, MD. (2001).

14. R. F. Lyon, A computational model of filtering, detection, and compression in the cochlea in *ICASSP ’82. IEEE International Conference on Acoustics, Speech, and Signal Processing*, (Institute of Electrical and Electronics Engineers, 1982), pp. 1282–1285.

15. R. F. Lyon, Cascades of two-pole–two-zero asymmetric resonators are good models of peripheral auditory function. *J. Acoust. Soc. Am.* **130**, 3893–3904 (2011).

16. I. C. Bruce, Y. Erfani, M. S. A. Zilany, A phenomenological model of the synapse between the inner hair cell and auditory nerve: Implications of limited neurotransmitter release sites. *Hear. Res.* **360**, 40–54 (2018).

17. M. S. A. Zilany, I. C. Bruce, L. H. Carney, Updated parameters and expanded simulation options for a model of the auditory periphery. *J. Acoust. Soc. Am.* **135**, 283–286 (2014).

18. M. S. A. Zilany, I. C. Bruce, P. C. Nelson, L. H. Carney, A phenomenological model of the synapse between the inner hair cell and auditory nerve: Long-term adaptation with power-law dynamics. *J. Acoust. Soc. Am.* **126**, 2390–2412 (2009).

19. R. Meddis, Auditory-nerve first-spike latency and auditory absolute threshold: A computer model. *J. Acoust. Soc. Am.* **119**, 406–417 (2006).

20. R. Meddis, *et al.*, "A computer model of the auditory periphery and its application to the study of hearing" in *Basic Aspects of Hearing*, B. C. J. Moore, R. D. Patterson, I. M. Winter, R. P. Carlyon, H. E. Gockel, Eds. (Springer, New York, NY, 2013), pp. 11–20.

21. M. A. Steadman, C. J. Sumner, Changes in neuronal representations of consonants in the ascending auditory system and their role in speech recognition. *Front. Neurosci.* **12**, 671 (2018).

22. C. J. Sumner, L. P. O'Mard, E. A. Lopez-poveda, R. Meddis, A nonlinear filter-bank model of the guinea-pig cochlear nerve: Rate responses. *J. Acoust. Soc. Am.* **113**, 3264 (2003).

23. R. Meddis, L. P. O'Mard, E. A. Lopez-Poveda, A computational algorithm for computing nonlinear auditory frequency selectivity. *J. Acoust. Soc. Am.* **109**, 2852–2861 (2001).

24. C. J. Sumner, E. A. Lopez-Poveda, L. P. O'Mard, R. Meddis, A revised model of the inner-hair cell and auditory-nerve complex. *J. Acoust. Soc. Am.* **111**, 2178 (2002).

25. M. B. Sachs, P. J. Abbas, Rate versus level functions for auditory-nerve fibers in cats: Bandlimited noise bursts. *J. Acoust. Soc. Am.* **64**, S135–S135 (1978).

26. H. S. Colburn, L. H. Carney, M. G. Heinz, Quantifying the information in auditory-nerve responses for level discrimination. *J. Assoc. Res. Otolaryngol.* **4**, 294–311 (2003).

27. D. A. Depireux, J. Z. Simon, D. J. Klein, S. A. Shamma, Spectro-temporal response field characterization with dynamic ripples in ferret primary auditory cortex. *J. Neurophysiol.* **85**, 1220–1234 (2001).

28. J. Friedman, T. Hastie, R. Tibshirani, Regularization paths for generalized linear models. *J. Stat. Softw.* **33**, 1–3 (2010).

29. J. Sohl-Dickstein, B. Poole, S. Ganguli, Fast large-scale optimization by unifying stochastic

gradient and quasi-Newton methods. *arXiv Prepr. arXiv1311.2115* (2013).

30. X. Glorot, Y. Bengio, Understanding the difficulty of training deep feedforward neural networks in *Proc. AISTATS*, (2010), pp. 249–256.

Legends for Audio Files

Audio File S1. All sound stimuli in natural sound dataset 1 combined in a single file.

Audio File S2. All sound stimuli in natural sound dataset 2 combined in a single file.

Audio File S3. All sound stimuli of the DRC dataset combined in a single file.
